# Hippo Pathway–YAP/TAZ Signaling: Molecular Mechanisms, Biological Function, Diseases, and Therapeutic Targets

**DOI:** 10.1002/mco2.71027

**Published:** 2026-09-23

**Authors:** Xiaodan Qu, Zhan‐you Wang

**Affiliations:** ^1^ Key Laboratory of Major Chronic Diseases of Nervous System of Liaoning Province Health Sciences Institute of China Medical University Shenyang PR China

**Keywords:** drug resistance, fibrosis, Hippo pathway, PROTAC, TEAD inhibitor, YAP/TAZ–TEAD

## Abstract

The Hippo pathway and its downstream effectors Yes‐associated protein/transcriptional coactivator with PDZ‐binding motif‐TEA domain transcription factor (YAP/TAZ–TEAD) play critical roles in organ‐size control, tissue homeostasis, regeneration, and stem‐cell biology. Their aberrant activation drives malignancies, fibrosis, cardiovascular disease, and immune dysregulation. Once regarded undruggable, the YAP/TAZ–TEAD complex has become one of the most actively pursued target classes in oncology and beyond. In this review, we delineate the biological functions of the Hippo–YAP/TAZ–TEAD axis and integrate its pathway physiology with the structural basis of druggability, centered on the Ω‐loop surface pocket and the buried palmitoylation‐binding pocket (PBP) of TEAD, whose distinct geometries dictate the pharmacophoric requirements, paralog selectivity, and resistance liabilities of current agents. We then systematically compare mechanism‐guided therapeutic modalities, encompassing direct protein–protein interaction disruptors, covalent and noncovalent PBP inhibitors, proteolysis‐targeting chimeras (PROTACs), and cofactor‐interface modulators. We also analyze the upstream kinase (MST1/2, LATS1/2) modulators, direct YAP/TAZ inhibitors and degraders, and emerging gene‐, RNA‐, antibody‐, and cell‐based therapies. We critically evaluate preclinical and early clinical performance across oncology, fibrosis, immunology, and regenerative medicine, distinguishing robust proof‐of‐concept from clinically meaningful benefit. We further dissect resistance mechanisms, on‐target safety concerns, and the therapeutic‐window limitations of pan‐TEAD inhibition. Ultimately, we outline rational combination strategies, biomarker‐guided patient selection, and future directions for paralog‐selective and tissue‐restricted Hippo‐targeted therapeutics.

## Graphical abstract

The Hippo–YAP/TAZ–TEAD pathway integrates mechanical and biochemical cues to govern organ growth, regeneration, cancer, and fibrosis. This review dissects pathway physiology, TEAD structural pharmacology and ligandable pockets, and therapeutic strategies spanning palmitoylation‐pocket inhibitors, PROTAC degraders, and gene/RNA therapies, highlighting biomarker‐guided translation and resistance mechanisms.

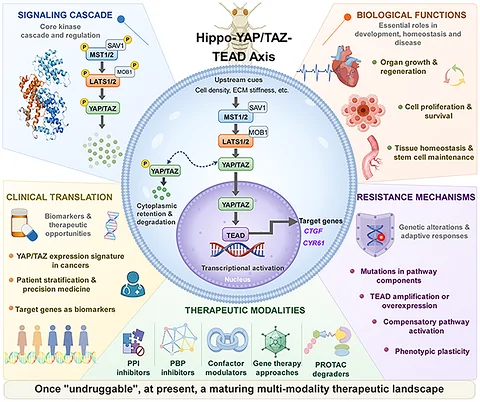

## 1 Introduction

The Hippo signaling pathway, first identified in *Drosophila* and highly conserved in mammals, has emerged as a central regulator of organ size, tissue homeostasis, regeneration, and stem‐cell biology [1, 2]. Its core output (the transcriptional coactivators Yes‐associated protein /transcriptional coactivator with PDZ‐binding motif [YAP/TAZ] acting through the TEA domain transcription factor [TEAD] family of transcription factors) integrates biochemical, mechanical, and metabolic cues to orchestrate cell proliferation, survival, and fate decisions. Dysregulation of this axis drives a broad spectrum of human diseases, including cancer, fibrosis, cardiovascular disease, and immune disorders. Elevated nuclear YAP expression independently predicts adverse clinical outcomes across tumor types. Once considered undruggable, the Hippo–YAP/TAZ–TEAD axis has rapidly matured into a clinically tractable target class, exemplified by objective clinical responses to the TEAD palmitoylation inhibitor VT3989 in neurofibromatosis type 2 (NF2)‐deficient mesothelioma. This review provides a mechanistically integrated synthesis of the biological functions, molecular mechanisms and therapeutic targeting of the Hippo–YAP/TAZ–TEAD pathway, and critically appraises the translational landscape from small molecules to gene‐, RNA‐, antibody‐, and cell‐based modalities.

This review progresses from biological function and molecular mechanism to therapeutic targeting and clinical translation. We summarize the biological functions of the Hippo–YAP/TAZ–TEAD axis in organ‐size control, tissue repair, stem‐cell regulation, and mechanotransduction, together with its disease associations (Section 2). We then integrate the pathway physiology with the structural basis of the YAP–TEAD complex and its long‐standing “undruggability” (Section 3). Subsequently, we systematically compare the mechanism‐based design features of TEAD‐directed small molecules and discuss the upstream kinase modulators, direct YAP/TAZ inhibitors and degraders, and gene‐, RNA‐, antibody‐, and cell‐based therapeutic modalities, highlighting their differentiated therapeutic advantages, inherent liabilities and most appropriate disease indications (Section 4). Building on this foundation, we critically appraise preclinical and clinical progress across oncology, fibrosis, immunology, and regenerative medicine (Section 5). Moreover, we analyze the molecular mechanisms of intrinsic and acquired resistance together with on‐target safety liabilities, in order to define the therapeutic window of pan‐TEAD inhibition versus paralog‐selective and tissue‐targeted approaches (Section 6). Finally, we articulate design principles for next‐generation therapeutics, including strategies for tissue‐specific delivery, biomarker‐driven patient stratification and rational combination therapies.

## 2 Biological Functions of the Hippo–YAP/TAZ–TEAD Axis

The Hippo–YAP/TAZ–TEAD axis orchestrates a broad spectrum of biological processes that are essential for embryonic development, adult tissue homeostasis, and regeneration. Understanding these physiological functions is a prerequisite for rational therapeutic targeting, because they define both the disease mechanisms driven by pathway dysregulation and the on‐target liabilities of pathway inhibition. In this section, we systematically delineate the biological functions of the axis, including organ‐size control, tissue repair and regeneration, stem‐cell regulation, and mechanotransduction, and summarize how its dysregulation contributes to cancer, fibrotic disease, cardiovascular disorders, and immune dysregulation.

### 2.1 Organ‐Size Control and Tissue Homeostasis

The Hippo signaling pathway was initially discovered in *Drosophila* through genetic screening in the early 2000s, named for the “hippopotamus‐like” overgrowth phenotype caused by loss‐of‐function mutations [1, 2]. In mammals, this highly conserved kinase cascade consists of core upstream kinase complexes, including mammalian sterile 20‐like kinase 1/2 (MST1/2), Salvador family WW domain‐containing protein 1 (SAV1), large tumor suppressor kinase 1/2 (LATS1/2), MOB kinase activator 1 (MOB1), and downstream effectors YAP and transcriptional coactivator with TAZ. When the Hippo pathway is activated, MST1/2 phosphorylates and activates LATS1/2, which further phosphorylates YAP at Ser127 and TAZ at Ser89, leading to their cytoplasmic sequestration via binding to 14‐3‐3 proteins and subsequent ubiquitin‐proteasomal degradation. When the pathway is inhibited, dephosphorylated YAP/TAZ translocate into the nucleus, bind to the N‐terminal domains of TEAD transcription factors (TEAD1–4), recruit transcriptional coactivator complexes, and drive expression of downstream target genes [3]. Under homeostatic conditions, this YAP/TAZ–TEAD axis governs three fundamental processes including organ‐size control, tissue homeostasis and damage repair, and stem‐cell regulation. In organ‐size control, the pathway precisely regulates the balance between cell proliferation and apoptosis to determine final organ size of liver, heart, and eye [1]. Studies have shown that liver‑specific knockout of *Mst1/2* leads to a three‑ to fourfold increase in liver size, whereas *LATS1* overexpression significantly reduces organ volume [4].

### 2.2 Tissue Repair and Regeneration

In tissue homeostasis and damage repair, YAP/TAZ function as sensors of cell density and the mechanical microenvironment. Increased cell–cell contact activates Hippo signaling, causing YAP/TAZ phosphorylation and cytoplasmic retention to restrain proliferation [5, 6, 7]. Upon tissue injury, reduced cell density and mechanical stress changes resulting from extracellular matrix (ECM) remodeling cause transient Hippo pathway inhibition. This allows YAP/TAZ to enter the nucleus and activate downstream proliferation‑ and regeneration‑related genes, promoting stem‐cell activation, progenitor cell proliferation and epithelial cell migration to accelerate damage repair [8, 9, 10]. Furthermore, YAP/TAZ transient activation plays an irreplaceable role in injury repair models across different organs. After partial hepatectomy, YAP activation in hepatocytes is a necessary condition for initiating liver regeneration, and hepatocyte‑specific YAP knockout reduces liver regenerative capacity by more than 70% [4]. Following myocardial infarction (MI), transient YAP activation in cardiomyocytes (CMs) promotes CM proliferation, reduces infarct size and improves cardiac function [11, 12, 13]. During skin injury, YAP activation in keratinocytes promotes re‑epithelialization and accelerates wound healing [14, 15]. After intestinal injury, YAP activation promotes intestinal stem‐cell proliferation and repairs the intestinal barrier [16, 17, 18]. Notably, this activation is strictly spatiotemporally regulated. YAP/TAZ return to the cytoplasm after repair is completed, whereas sustained activation leads to tissue fibrosis and tumorigenesis [19, 20]. Remarkably, YAP–TEAD signaling axis can further amplify regenerative signals by regulating the polarization of tissue‑resident macrophages and ECM remodeling, thereby participating in the regulation of the injury microenvironment [19, 21, 22]. Additionally, YAP agonists significantly promote muscle regeneration and skin wound healing in aged mice, demonstrating the great potential of YAP–TEAD modulators in regenerative medicine [23, 24].

### 2.3 Stem‐Cell Regulation and Pluripotency

In stem‐cell regulation, YAP/TAZ are abundantly expressed in embryonic stem cells, adult stem cells, and cancer stem cells. They maintain the self‑renewal capacity and multidirectional differentiation potential of stem cells by regulating the expression of stemness‑related genes [25, 26, 27, 28, 29]. In embryonic stem cells, YAP–TEAD transcriptional complexes directly activate OCT4 to drive the subsequent expansion of pluripotent cells. Meanwhile, YAP cooperates with master transcription factors Nanog, *SOX2*, and *OCT4* to maintain pluripotency to establish superenhancers that reinforce the pluripotency network [25, 26]. In adult tissue‐resident stem cells such as hair follicle bulge stem cells, nuclear YAP/TAZ activity is correlated with both proliferative capacity and multipotent differentiation potential [27]. Notably, in cancer stem cells, YAP/TAZ directly transcriptionally regulate stemness‐related proteins including OCT4, SOX2, and SOX9, with TAZ gain‐of‐function endowing noncancer stem cells with self‐renewal abilities and tumorigenicity [28, 29]. Moreover, the phase separation‐mediated formation of YAP‐bound superenhancers represents an emerging mechanism coordinating stem‐cell transcriptional programs [30].

### 2.4 Mechanotransduction and Metabolic Regulation

Beyond cell‐intrinsic cues, YAP/TAZ activity is exquisitely controlled by the mechanical and metabolic state of the tissue microenvironment. The small GTPase Ras‐related protein Rap‐2 (RAP2) functions as a central integrator of cytoskeletal signals, transducing low matrix stiffness, energy stress, and serum deprivation into LATS1/2 activation through mitogen‐activated protein kinase kinase kinase kinases (MAP4Ks) and MST1/2, whereas Ras homolog family member A (RhoA)‐driven actin polymerization antagonizes this module to sustain YAP/TAZ nuclear localization [31]. Proteostatic stress has recently been identified as an additional upstream input. Proteasome inhibition triggers RAP2 ubiquitination and inactivation, disrupting the RAP2–MAP4K–NF2–LATS1/2 cascade and thereby activating YAP/TAZ, which in turn promotes tumor‐cell survival and resistance to proteasome inhibitors [32]. Metabolic cues likewise converge on the pathway. Mevalonate‐derived cholesterol stabilizes TAZ in hepatocytes by suppressing beta‐transducin repeat‐containing protein (β‐TrCP)‐mediated proteasomal degradation, coupling nutrient availability to fibrotic progression in nonalcoholic steatohepatitis (NASH) [33], and under hypoxia, nucleus‐translocated glucokinase functions as a protein kinase that phosphorylates and stabilizes TAZ to promote tumor growth [34]. At the transcriptional level, liquid–liquid phase separation (LLPS) of YAP into nuclear condensates organizes superenhancer assembly and coordinates stem‐cell and proliferative transcriptional programs. Collectively, these findings establish YAP/TAZ as central integrators of mechanical, metabolic, and proteostatic signals, a property that underlies both their physiological versatility and their pathological exploitation.

### 2.5 Roles in Cancer

**FIGURE 1:**
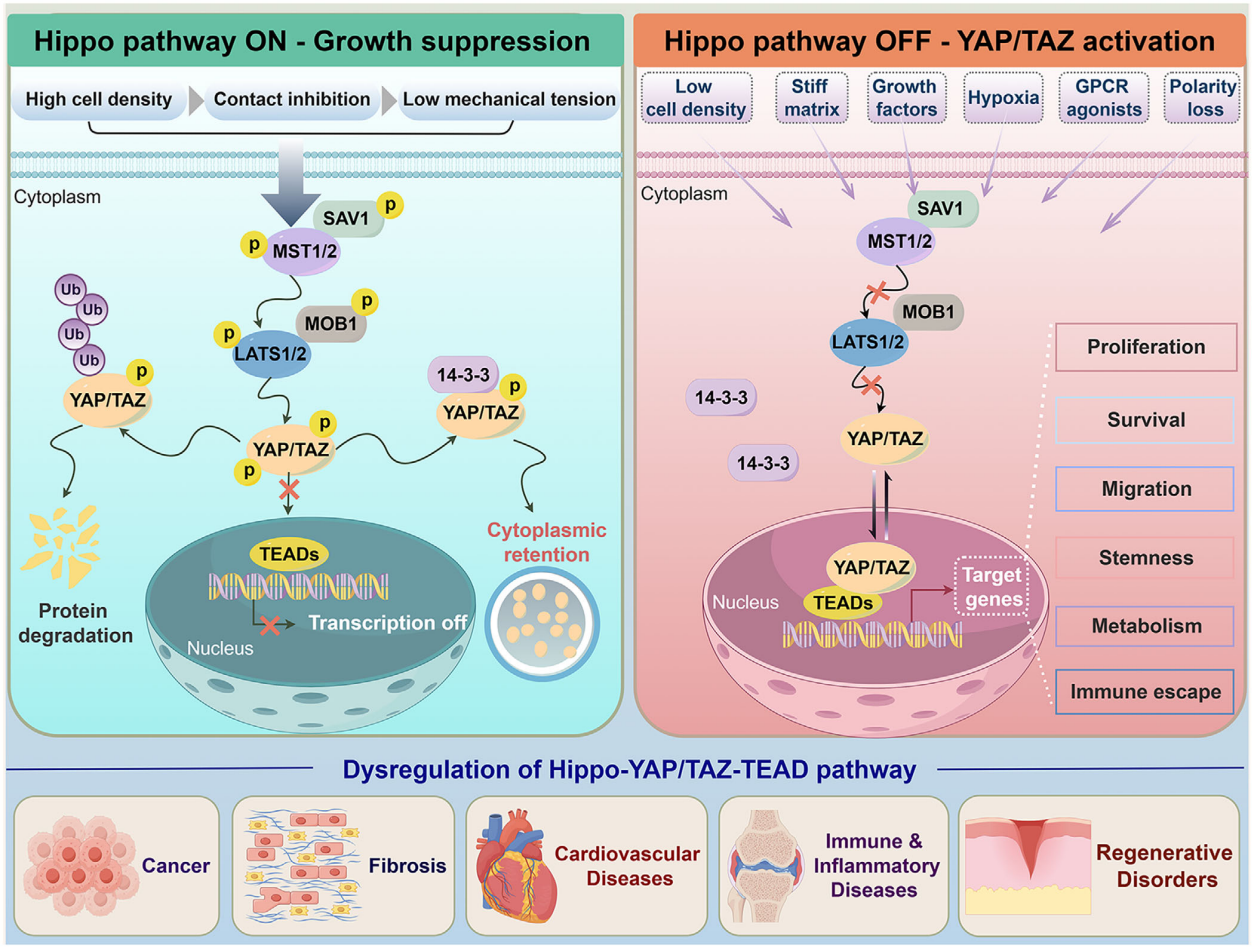
Regulation of the Hippo‐YAP/TAZ signaling pathway under physiological and pathological conditions. Under conditions that activate the Hippo pathway (Left), MST1/2, together with the scaffold protein SAV1, phosphorylate and activate LATS1/2. Activated LATS1/2 subsequently phosphorylate YAP/TAZ, creating binding sites for 14‐3‐3 proteins, which sequester YAP/TAZ in the cytoplasm and promote their ubiquitin‐proteasome‐mediated degradation. In the nucleus, TEAD transcription factors remain transcriptionally inactive due to the absence of coactivators, leading to silencing of downstream target genes. Cells maintain quiescence, differentiation or apoptosis. In contrast, when the Hippo pathway is inactivated (Right), the MST1/2‐LATS1/2 kinase cascade is suppressed. Unphosphorylated YAP/TAZ translocate into the nucleus, where they bind TEAD family transcription factors, initiating transcriptional programs that promote proliferation, survival, migration, stemness, metabolic reprogramming, and immune escape. In addition, sustained Hippo‐OFF (YAP/TAZ hyperactivation) is associated with multiple human diseases, including cancer (mesothelioma, hepatocellular carcinoma, and *KRAS*‐mutant lung cancer), fibrosis (idiopathic pulmonary fibrosis, liver fibrosis, cardiac fibrosis, renal fibrosis, keloids), cardiovascular diseases (myocardial hypertrophy, heart failure), immune and inflammatory diseases (rheumatoid arthritis, multiple sclerosis), and pathological organ overgrowth or impaired regeneration (autosomal dominant polycystic kidney disease, impaired wound healing). This signaling switch integrates diverse upstream inputs, including mechanical stress, cell polarity, growth factors, GPCR signaling, and oncogenic signals, to maintain tissue homeostasis; its aberrant activation represents a core molecular event underlying these pathological conditions.

The transcriptional program driven by YAP/TAZ–TEAD encompasses genes promoting (1) cell proliferation, including cellular myelocytomatosis (*c‐Myc*), cyclin D1 (*CCND1*), forkhead box M1 (*FOXM1*), and baculoviral IAP repeat containing 5 (*BIRC5*, *survivin*) [35]; (2) survival and antiapoptosis, such as myeloid cell leukemia 1 (*MCL1*) [36] and FLICE‐like inhibitory protein (*CFLAR*, *c‐FLIP*) [37], ankyrin repeat domain 1 (*ANKRD1*) [38]; (3) migration and invasion, including connective tissue growth factor (*CTGF*, *CCN2*) [39], cysteine‐rich angiogenic inducer 61 (*CYR61*, *CCN1*) [40], amphiregulin (*AREG*) [41], and matrix metallopeptidases (*MMPs*) [42]; (4) stemness and pluripotency, exemplified by SRY‐box transcription factor 2 (*SOX2*), octamer‐binding transcription factor 4 (*OCT4*, *POU5F1*), Nanog homeobox (*NANOG*) [43]; (5) metabolic reprogramming, involving glucose transporter 3 (*GLUT3*, *SLC2A3*), hexokinase 2 (*HK2*), and lactate dehydrogenase A (*LDHA*) [44, 45]. Aberrant activation of this program is linked to malignancies, fibrosis, cardiovascular remodeling, immune and inflammatory disorders and impaired regeneration, making YAP/TAZ–TEAD a highly pursued target. The core regulatory logic of the Hippo pathway is illustrated in Figure 1, which delineates the molecular events distinguishing Hippo‐ON and Hippo‐OFF states. In oncology, aberrant activation of YAP/TAZ is frequently observed in human solid tumors, where it serves as a critical driver of malignant progression [46, 47]. A meta‐analysis of over 6700 patients from 53 independent studies demonstrates that elevated nuclear YAP1 expression represents an independent prognostic factor for adverse clinical outcomes, conferring increased mortality, recurrence, and treatment resistance [46]. In triple‐negative breast cancer (TNBC), aberrant YAP activation promotes cancer stem‐cell maintenance and chemoresistance. In non–small‐cell lung cancer (NSCLC), high nuclear YAP expression correlates with the epidermal growth factor receptor (EGFR)‐tyrosine kinase inhibitor (TKI) resistance and metastasis [47, 48]. The oncogenic potential of YAP/TAZ is further amplified through their function as mechanotransducers in the stiff tumor stroma, where integrin‐actomyosin signaling induces their nuclear translocation, creating a feed‐forward loop that drives cancer‐associated fibroblast (CAF) activation, collagen crosslinking, epithelial–mesenchymal transition (EMT), angiogenesis, and immune evasion [47, 49]. Additionally, NF2 mutations, common in meningiomas and mesotheliomas, lead to constitutive YAP/TAZ activation, promoting aerobic glycolysis and suppressing antitumor immunity [50, 51]. Importantly, oncogenic signaling pathways such as rat sarcoma (RAS)/mitogen‐activated protein kinases (MAPK), phosphatidylinositol 3‐kinase (PI3K)/protein kinase B (PKB/AKT), and Wnt/β‐catenin also converge on YAP/TAZ activation [47, 52, 53, 54, 55]. Oncogenic KRAS activates YAP/TAZ via OTUB2‐mediated deubiquitination and SUMOylation independently of Hippo signaling, positioning YAP as a pivotal transcriptional switch downstream of KRAS‐MAPK to drive neoplastic proliferation [47, 56]. In phosphatase and tensin homolog (PTEN)‐loss sarcomas, PI3K activation leads to LATS1/2‐mediated YAP/TAZ regulation [53, 57]. Furthermore, Wnt signaling engages YAP/TAZ through noncanonical frizzled/receptor tyrosine kinase‑like orphan receptor‐G protein subunit alpha 12/13‐ras homolog gene family (FZD/ROR‐Gα12/13‐Rho) pathways, while reciprocal regulation occurs via the β‐catenin destruction complex, enabling cooperative transcriptional control by YAP–TEAD and β‐catenin‐T‐cell factor (TCF) complexes [54, 55]. Collectively, these alterations position YAP/TAZ as central integrators of biochemical, mechanical and metabolic signals that orchestrate tumor invasion, metastasis, and therapeutic resistance.

### 2.6 Roles in Fibrotic Diseases

In fibrotic diseases, sustained YAP/TAZ activation is a hallmark of fibrotic disorders across multiple organs, including the heart, lung, liver, kidney, and skin [21]. Mechanistically, YAP/TAZ serve as critical mechanotransducers that drive fibroblast‐to‐myofibroblast transition, promote excessive ECM deposition, and suppress matrix degradation, thereby initiating and perpetuating fibrosis through mechanically self‐reinforcing positive feedback loops [47, 58]. Upon tissue injury, increased ECM stiffness activates YAP/TAZ in fibroblasts via integrin‐mediated cytoskeletal rearrangement and RhoA/Rho‐associated protein kinase (ROCK)‐dependent pathways, independent of canonical Hippo signaling [59, 60, 61]. Nuclear YAP/TAZ subsequently upregulate profibrotic genes including CTGF, collagen type I, fibronectin, and plasminogen activator inhibitor‐1 (PAI‐1), while simultaneously promoting myofibroblast contractility and survival [62, 63]. This excessive ECM accumulation further increases tissue stiffness, establishing a feed‐forward amplification loop that perpetuates fibrogenesis [63]. In the lung, YAP/TAZ sustain a profibrotic transcriptional program in both fibroblasts and alveolar epithelial type 2 (AT2) cells, with nuclear YAP levels elevated in idiopathic pulmonary fibrosis (IPF) patients and correlating positively with fibrosis severity and disease progression [64]. In the liver, hepatic stellate cells (HSCs) undergo YAP‐dependent activation into myofibroblasts, with Hedgehog‐YAP‐regulated glutaminolysis sustaining their metabolic reprogramming [64]. In the kidney, YAP mediates TGF‐β1‐induced fibroblast activation through direct interaction with Smad2/3 and *CTGF*‐dependent suppression of Smad7, while matrix stiffness induces YAP/TAZ nuclear translocation through ROCK1 [65]. Collectively, these findings establish sustained YAP/TAZ activation as a central hub integrating mechanical, biochemical, and metabolic signals to orchestrate organ fibrosis, positioning YAP/TAZ as promising therapeutic targets.

### 2.7 Roles in Cardiovascular Disease

In cardiovascular disease, YAP/TAZ play important roles in myocardial hypertrophy, MI, hypertension, and atherosclerosis [66]. In response to pressure overload (PO), a consequence of hypertension or aortic stenosis, YAP/TAZ serve as critical mechanotransducers that coordinate adaptive and maladaptive cardiac remodeling [66]. Acute activation of YAP in CMs promotes cell survival and regeneration; however, sustained YAP activation under chronic PO conditions triggers CM dedifferentiation and heart failure progression through a detrimental YAP–TEAD1 positive feedback loop involving oncostatin M (OSM) signaling [67]. In PO‐induced hypertrophy model, CM‐specific YAP knockout reduces cardiac YAP expression and significantly attenuates pathological hypertrophy while preserving cardiac function [68]. Following MI, YAP/TAZ exhibit cell‐type‐specific functions. In CMs, YAP activation enhances survival and regenerative capacity; conversely, in cardiac fibroblasts, YAP/TAZ drive myofibroblast differentiation, excessive ECM deposition, and proinflammatory cytokine secretion, exacerbating fibrotic remodeling [69]. Fibroblast‐specific deletion of Yap/Taz using *Col1a2^Cre(ER)T^* mice reduces post‐MI fibrotic, fibroinflammatory responses and macrophage infiltration, improving cardiac function [70]. In macrophages, YAP/TAZ promote proinflammatory polarization through histone deacetylase 3 (HDAC3)‐nuclear receptor corepressor 1 (NCoR1)‐mediated repression of arginase‐1 and activation of interleukin‐6, thereby impairing reparative responses and amplifying adverse remodeling [71]. In the vasculature, disturbed flow activates endothelial YAP/TAZ, promoting glycolysis, inflammation, and dysfunction, whereas atheroprotective unidirectional shear stress suppresses YAP/TAZ activity [66]. In diabetic cardiomyopathy, chronic hyperglycemia and mechanical stress synergistically activate YAP/TAZ, exacerbating myocardial fibrosis, hypertrophy and mitochondrial dysfunction [66]. Collectively, these findings position YAP/TAZ as central integrators of hemodynamic, metabolic, and inflammatory signals in cardiovascular disease, with their cell‐type‐specific functions dictating divergent outcomes, cardioprotective in CMs but pathogenic in fibroblasts, macrophages, and endothelial cells, necessitating targeted therapeutic strategies.

### 2.8 Roles in Immune and Inflammatory Disorders

In immune and inflammatory disorders, YAP/TAZ regulate both innate and adaptive immunity, and contribute to autoimmune disorders including rheumatoid arthritis (RA), multiple sclerosis (MS), and inflammatory bowel disease [72]. In the innate immune system, YAP/TAZ modulate macrophage polarization, dendritic cell activation and pyrin domain‐containing protein 3 (NLRP3) inflammasome assembly [73]. Mechanistically, YAP promotes NLRP3 inflammasome activation by physically interacting with NLRP3 and stabilizing it through blockade of K27‐linked polyubiquitination and proteasomal degradation, thereby amplifying inflammatory responses in systemic inflammation, gout, colitis and sepsis [73]. In the adaptive immune system, YAP and TAZ exhibit opposing functions in T‐cell differentiation. While YAP is highly expressed in immunosuppressive regulatory T cells (Tregs) and reinforces their functions, TAZ inhibits Treg differentiation and promotes the development of inflammatory T helper 17 (Th17) cells, creating a dynamic balance that dictates immune tolerance versus autoimmunity [47, 74, 75]. In RA, YAP/TAZ activity is markedly elevated in fibroblast‐like synoviocytes (FLS) driven by synergistic inflammatory cytokines (TNF and IL‐17) and synovial stiffening [76]. This establishes a self‐perpetuating loop wherein YAP/TAZ induce FLS invasive phenotype, synovial angiogenesis and Treg/Th17 imbalance, with Janus kinase (JAK) inhibitors such as baricitinib ameliorating RA partly by modulating YAP activity in FLS [77]. In MS and experimental autoimmune encephalomyelitis (EAE), astrocytic YAP functions as a critical neuroprotective regulator. Astrocyte‐specific YAP knockout significantly exacerbates EAE severity, accelerates demyelination, and increases neuronal loss [72]. Mechanistically, YAP prevents neuroinflammation and demyelination by transcriptionally upregulating cholesterol synthesis genes such as HMG‐CoA synthase 1 (HMGCS1) to support myelin maintenance, and enhancing TGF‐β signaling to suppress astrocyte activation [72]. Collectively, YAP/TAZ integrates inflammatory, metabolic, and mechanical signals in immune regulation, with dual roles as both proinflammatory amplifiers and immunosuppressive modulators, offering promising therapeutic targets for autoimmune and inflammatory disorders.

### 2.9 Spatiotemporal Dynamics of YAP/TAZ Activation: Implications for Therapy

A recurring theme across these biological contexts is that the outcome of YAP/TAZ modulation is dictated by the duration, magnitude, and tissue specificity of pathway activation. Transient, tightly controlled activation drives physiological repair, whereas sustained activation underlies fibrosis and tumorigenesis [19, 20]. Inhibition of YAP/TAZ–TEAD signaling is relevant for cancer and fibrotic disease, whereas transient pathway activation is relevant for regenerative indications. Pharmacological Hippo‐kinase inhibition that augments liver, intestinal, periodontal, and corneal endothelial regeneration [78, 79, 80, 81]. Understanding the spatiotemporal dynamics of YAP/TAZ activation is therefore a prerequisite for defining the therapeutic window of any Hippo pathway‐directed agent.

## 3 Molecular Mechanisms and Structural Basis of Hippo–YAP/TAZ–TEAD Signaling

Although the therapeutic potential of the YAP–TEAD signaling axis as a drug target has been widely recognized, this target was considered “undruggable” for a long time, mainly due to three fundamental challenges. First, the YAP–TEAD interaction interface spans approximately 3500 square angstroms (Å^2^) and exhibits a relatively flat topography devoid of deep pockets, rendering it inherently refractory to disruption by conventional small molecules that require well‐defined binding clefts [82, 83]. Second, the YAP–TEAD complex harbors three independent protein–protein interaction (PPI) interfaces that together confer high‐affinity binding and greatly impede small‐molecule disruption [84]. Finally, given the indispensable roles of YAP/TAZ in normal tissue homeostasis, systemic inhibition may lead to serious adverse reactions including nephrotoxicity and intestinal damage, resulting in a narrow therapeutic window [35, 85]. This impasse was transformed by the discovery in 2018 that TEAD proteins undergo autopalmitoylation and harbor a deep, central palmitoylation‐binding pocket (PBP). This pocket, together with a more surface‐exposed Ω‐loop pocket on the YAP‐binding face, provided two genuinely druggable sites and catalyzed an unprecedented expansion of pharmacological modalities. These include direct YAP‐TEAD PPI disruptors, covalent and noncovalent PBP inhibitors, TEAD‐targeted proteolysis‐targeting chimeras (PROTACs) and molecular glue degraders, and cofactor‐interface modulators. Clinical translation has followed rapidly. The TEAD PBP inhibitor VT3989 has yielded objective responses in patients with NF2‐deficient mesothelioma, whereas first‐in‐human trials of IAG933 and IK‐930 have been halted, underscoring the urgent need for a mechanistically integrated synthesis that discriminates successful strategies from those that fall short and explains the underlying reasons.

### 3.1 Pathway Physiology: The Core Kinase Cascade and Upstream Regulation

The core kinase cassette consists of MST1/2 complexed with SAV1, which phosphorylates and activates the LATS1/2–MOB1 complex, which in turn phosphorylates YAP/TAZ to drive 14‐3‐3‐mediated cytoplasmic sequestration and β‐TrCP‐dependent proteasomal degradation. This cassette is gated by an extensive upstream regulatory network. At the cell cortex, the tumor suppressor NF2 (Merlin) and the KIBRA–AMOT complex recruit and activate LATS1/2, whereas the striatin‐interacting phosphatase and kinase (STRIPAK) complex dephosphorylates and inactivates MST1/2. Cell–cell contact, apicobasal polarity complexes and GPCR–RhoA–actomyosin signaling provide additional inputs that render the pathway exquisitely sensitive to tissue architecture and mechanical state [5, 6, 7]. Recent work has substantially expanded this regulatory landscape. The microtubule affinity‐regulating kinases MARK2 and MARK3 directly phosphorylate NF2 and YAP/TAZ, functionally reversing the tumor‐suppressive output of LATS1/2; paralog cotargeting screens identified MARK2/3 as absolute catalytic requirements for YAP/TAZ function in diverse carcinomas and sarcomas, and their inhibition restores Hippo‐mediated tumor suppression and regresses established tumors in vivo [86]. The small GTPase RAP2 transduces cytoskeletal and proteostatic stress into LATS1/2 activation via MAP4Ks [31, 32]. Beyond phosphorylation, YAP/TAZ activity is further controlled at the levels of nucleocytoplasmic trafficking, LLPS, and transcriptional condensate formation. Together, these mechanistic layers define multiple points of pharmacological intervention, including upstream kinases, the YAP/TAZ effectors themselves and the TEAD transcription factors.

To establish the structural basis for TEAD‐directed drug design, we outline the domain architecture of YAP and the four TEAD paralogs (TEAD1– TEAD4) and the topology of the YAP–TEAD interaction interface. And then present the two ligandable pockets (the surface‐exposed Ω‐loop pocket and the buried PBP) and examine the structural determinants of their ligandability and paralog selectivity.

### 3.2 Structural Features of YAP and TEAD Proteins and Key Amino‐Acid Residues

#### 3.2.1 Domain Architecture and Functional Sites of YAP Protein

**FIGURE 2:**
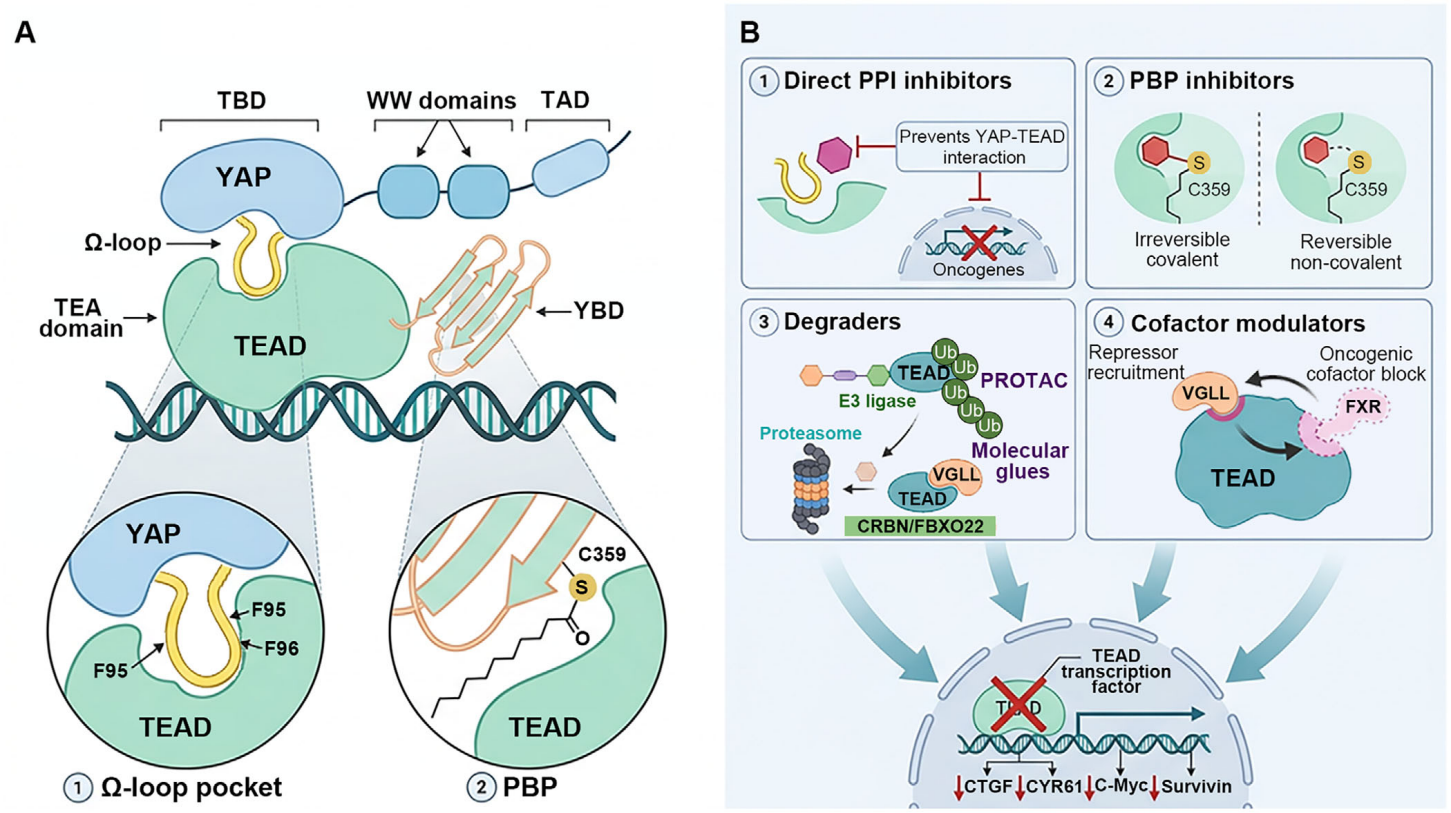
Structural basis of YAP–TEAD targeting and inhibition strategies. (A) Domain organization of the YAP–TEAD complex and key druggable pockets. YAP engages TEAD through its N‑terminal TEAD‑binding domain, which contains three interaction interfaces, with the Ω‑loop (Interface 3) serving as the dominant affinity driver. The Ω‑loop inserts into a deep hydrophobic pocket on the surface of the TEAD YAP‑binding domain (YBD), accompanied by the formation of a stable salt bridge and hydrogen‑bond network. Within the YBD, an internal cavity constitutes the PBP, where a conserved cysteine (TEAD1 C359) undergoes autopalmitoylation essential for TEAD stability. Adjacent cofactor‑interaction surfaces provide additional pharmacological sites. (B) Four mechanistically distinct classes of small‑molecule inhibitors: (i) Direct PPI inhibitors competitively occupy the Ω‑loop pocket to sterically block YAP binding. (ii) PBP inhibitors bind the PBP either through irreversible covalent engagement of the conserved cysteine or reversible noncovalent occupation, disrupting TEAD autopalmitoylation and folding. (iii) PROTACs and molecular glues induce targeted TEAD degradation by recruiting E3 ubiquitin ligases or enforce repressive complexes such as TEAD–VGLL4, thereby eliminating both canonical and scaffolding functions of TEAD. (iv) Cofactor interaction modulators allosterically promote the association of TEAD with transcriptional repressors or block oncogenic cofactor binding, switching the transcriptional output from activation to repression. All four strategies converge on silencing the oncogenic YAP/TAZ–TEAD transcriptional program, downregulating target genes including CTGF, CYR61, c‑Myc, survivin, and so on.

YAP functions as a transcriptional coactivator encoded by the human *YAP1* gene, with its major isoform comprising 504 amino‐acid residues organized into distinct functional modules [87, 88]. The N‐terminal region encompasses the TEAD‐binding domain (TBD) spanning residues 50–171, which serves as the critical interface for specific recognition of TEAD transcription factors and acts as the primary determinant of YAP transcriptional output. This domain contains three independent interaction interfaces [89, 90]. Interface 1, encompassing residues 52–58, forms an antiparallel β‐sheet structure that engages the TEAD surface through hydrogen bonding and van der Waals interactions. Core residues include H52, Q53, I54, V55, H56, V57, and R58 [89, 91]. Interface 2, spanning residues 61–73, contains the conserved leucine‐X‐X‐leucine‐phenylalanine (LXXLF) motif (L65, L68, and F69), forming an α‐helix that inserts into the α3/α4 hydrophobic groove of TEAD [91, 92]. Interface 3, comprising residues 86–100, forms the characteristic Ω‐loop structure representing the highest‐affinity binding region. Core residues include M86, R89, L91, S94, F95, and F96 [89, 92], among which F95 and F96 insert into the deep pocket of TEAD through hydrophobic interactions and are critical for the interaction (Figure 2A). Furthermore, adjacent to the TBD resides the 14‐3‐3 binding motif spanning residues 122–131 with the sequence HVRAHSSPASL, which contains the critical phosphorylation site S127 [93]. Upon Hippo pathway activation, LATS1/2 kinases catalyze phosphorylation at S127, thereby promoting YAP association with 14‐3‐3 adaptor proteins and consequent cytoplasmic sequestration [94]. Besides, the central portion of YAP contains two tandem WW domains spanning residues 119–162 and 182–222, which specifically recognize and bind partner proteins harboring PPXY motifs [95]. The C‐terminal transcriptional activation domain (TAD) encompasses residues 450–504 and functions to recruit transcriptional coactivator complexes to drive expression of downstream target genes [96]. It contains another phosphorylation site S397 that mediates YAP ubiquitination and degradation [97].

#### 3.2.2 Structural Features of the TEAD Protein Family

The TEAD family serve as the principal transcriptional effectors downstream of YAP/TAZ signaling [98]. Each TEAD protein harbors an N‐terminal TEA domain spanning approximately residues 1–100 (comprising ∼68–75 amino acids), which functions as a highly conserved DNA‐binding domain that specifically recognizes and binds to the muscle‐CAT (M‐CAT) consensus motif (5′‐CATTCCT‐3′) in the genome to regulate target gene transcription [99]. This domain contains three α‐helices achieving sequence‐specific binding through insertion of helix 3 into the DNA recognition surface [100]. The C‐terminal region of TEAD proteins encompasses the YAP‐binding domain (YBD) spanning approximately residues 200–450. This domain adopts an immunoglobulin‐like β‐sandwich fold responsible for binding YAP/TAZ and is the main target for small‐molecule inhibitor development [101]. The YBD contains two key functional sites. The Ω‐loop binding pocket represents a deep hydrophobic cavity formed by conserved residues including K297, W299, F337, and Y429 in human TEAD4, which serves as the docking site for the YAP Ω‐loop motif [90]. Additionally, the PBP constitutes a distinct hydrophobic cavity of approximately 400–500 Å^3^ volume inside the YBD domain [102]. The core conserved cysteine residues (TEAD1 C359, TEAD2 C380, TEAD3 C371, TEAD4 C360) undergo autopalmitoylation (Figure 2A), which is essential for maintaining TEAD protein stability and folding, and is the main target for covalent inhibitors [103, 104].

#### 3.2.3 Molecular Features of the YAP–TEAD Interaction Interface

The binding of YAP to TEAD results from the synergistic action of multiple interfaces, with a total binding affinity in the nanomolar range (KD ≈ 40 nM) [101]. Among these interfaces, Interface 3 corresponding to the Ω‐loop region of YAP makes the predominant energetic contribution and has therefore emerged as the preferred architectural feature for small‐molecule inhibitor development [89]. The TEAD binding pocket surface exhibits significant electrostatic complementarity, with YAP R89 forming a stable salt bridge with TEAD D249, and YAP S94 engaging in hydrogen bonds with TEAD E240 and Y406, thereby stabilizing the complex structure [89]. The binding pockets of different TEAD subtypes are highly conserved, with only a few amino‐acid differences (such as H245 in TEAD1 whereas Q245 in TEAD2, TEAD3, and TEAD4), providing a structural basis for the development of subtype‐selective inhibitors [90].

### 3.3 Recently Discovered Druggable Sites

Recent structural biology investigations have further illuminated multiple new potential druggable sites within TEAD proteins. A landmark study in 2025 reported that in liver tumors, the YAP–TEAD complex can directly bind to the farnesoid X receptor (FXR) through a unique region of the TEAD YBD domain and recruit histone deacetylase 1 (HDAC 1) to inhibit FXR transcriptional activity, thereby promoting liver tumorigenesis [105]. This interaction interface is spatially segregated from the YAP binding site, providing a new direction for developing liver tumor‐specific TEAD inhibitors. Additionally, the transcriptional repressor Vestigial‐like family 4 (VGLL4) can bind to the YBD domain of TEAD through its second TEAD‐interacting domain (TDU2), competitively inhibiting the YAP–TEAD interaction [106]. Studies in 2025 found that small molecules can allosterically regulate TEAD conformation to enhance its binding affinity for VGLL4, thereby achieving pathway inhibition through a cofactor switch mechanism rather than direct YAP displacement [107].

### 3.4 Structural Determinants of Ligandability and Selectivity

The distinct topological features of Ω‑loop pocket and PBP dictate the design logic and pharmacological profiles of TEAD‑targeting agents. In detail, the Ω‑loop pocket is a shallow surface depression that requires inhibitors to precisely mimic the α‑helical turn and side‑chain orientation of the YAP Ω‑loop. High complementarity is achieved through three pharmacophoric elements, namely, two deep‑inserting hydrophobic residues (mimicking YAP F95/F96), a central salt‑bridge surrogate (R89–TEAD D249), and a flanking hydrogen‑bond network (S94–TEAD E240/Y406). This pocket is spatially segregated from the palmitoylation cavity; thus, occupancy does not directly perturb TEAD folding, which may confer a cleaner selectivity profile but limits the inhibitor to blocking only one of three YAP–TEAD interfaces, leaving interfaces 1 and 2 available for residual YAP binding or alternative cofactor engagement (e.g., PITX2, VGLL3). Moreover, the PBP forms a deeply buried, fully enclosed hydrophobic cavity. Its concave architecture enables high‑affinity binding of small molecules, while the conserved cysteine at the pocket entrance provides an anchor for covalent warheads. However, the > 70% sequence identity among TEAD1–4 means pan‑TEAD inhibition is almost inevitable unless subtle structural differences are exploited, such as the larger lower pocket volume in TEAD1, a nonconserved cysteine near the YAP interface unique to TEAD2, and amino‑acid variations that can be probed by atropisomeric compounds. The absolute dependence of TEAD stability on autopalmitoylation transforms the PBP from a passive lipid‑storage cavity into an Achilles’ heel, such that once the pocket is occupied, TEAD becomes conformationally destabilized and is degraded.

In summary, the flat YAP–TEAD interface, the Ω‐loop surface pocket and the buried PBP constitute three structurally distinct entry points, each imposing different pharmacophore, selectivity, and resistance constraints.

## 4 Therapeutic Targeting Strategies for the Hippo–YAP/TAZ–TEAD Axis

Following the discovery of druggable sites on TEAD, several mechanistically distinct classes of YAP–TEAD‐targeted therapeutics have been developed. We classify TEAD‐directed small molecules into four major categories based on the molecular site of intervention: (1) inhibitors directly disrupting the YAP–TEAD PPI interface; (2) inhibitors targeting the PBP of TEAD; (3) TEAD protein degraders, including PROTACs and molecular glues; and (4) strategies targeting alternative TEAD functional surfaces or cofactor interactions. Additionally, we discuss the upstream kinase modulators, direct YAP/TAZ inhibitors and degraders, and gene‐, RNA‐, antibody‐, and cell‐based modalities. As of 2026, more than 15 TEAD‐targeting agents have entered clinical development globally, with dozens more in preclinical optimization stages [85, 91, 108].

### 4.1 Direct YAP–TEAD PPI Inhibitors

These inhibitors predominantly target the Ω‐loop binding pocket on the TEAD surface, competitively obstructing YAP–TEAD interaction by mimicking the structure of the YAP Ω‐loop (Figure 2B). Initially, Verteporfin was the first discovered YAP–TEAD interaction inhibitor. This benzoporphyrin derivative has received Food and Drug Administration (FDA) approval for the treatment of age‐related macular degeneration (AMD) [109]. This compound inhibits YAP–TEAD interaction through multiple mechanisms. In one aspect, Verteporfin binds directly to YAP, inducing conformational changes that prevent YAP–TEAD complex formation [110]. Moreover, it induces YAP oligomerization, sequestering YAP in inactive complexes [111]. However, verteporfin's poor specificity beyond TEAD, its reliance on photoactivation for full potency, and its short in vivo half‐life approximately 5–6 h make it unsuitable as a systemic anticancer drug [111]. Multiple clinical trials have invested verteporfin in oncology, including NCT03033225 in 2017 (advanced pancreatic cancer in combination with chemotherapy) and NCT04590664 in 2021 (recurrent high‐grade EGFR‐mutated glioblastoma).

#### 4.1.1 Discovery Strategies and Mechanism Overview

The identification of direct YAP–TEAD PPI disruptors has been propelled by diverse screening and design approaches. Bioassay platforms have enabled high‐throughput evaluation. A fluorescence polarization (FP)‐based assay was established for the identification and evaluation of YAP–TEAD PPI inhibitors at the YAP Ω‐loop binding region. This FP method is reliable, robust, and economical for inhibitor assessment, and was validated using the patented small‐molecule Patent‐22 as a YAP–TEAD PPI inhibitor [112]. Computational and in silico methods have provided powerful frameworks for rational design. Advanced in silico techniques including free energy perturbation (FEP) calculations and molecular dynamics simulations in the early drug discovery of potent small‐molecule YAP–TEAD PPI disruptors, providing a computational framework for rational design of interface‐targeting compounds [113]. Structure‐based hit‐to‐lead optimization has yielded clinical candidates. Researchers at Novartis disclosed the first class of small molecules potently inhibiting the YAP–TEAD interaction by binding at one of the main interaction sites of YAP at the surface of TEAD. These inhibitors evolved from a weakly active virtual screening hit to high potency through structure‐based design, providing a path forward for pharmacological intervention in the Hippo pathway [83]. Subsequently, multiparameter optimization of a class of dihydrobenzofurane analogs, delivered advanced compounds that combined nanomolar cellular potency with a balanced ADME and off‐target profile. Crucially, efficacy of these orally bioavailable compounds in tumor‐bearing mice was demonstrated for the first time, validating the in vivo druggability of the Ω‐loop pocket and providing the chemical foundation for IAG933 [114].

#### 4.1.2 First‐in‐Class Clinical Candidate: IAG933

Through structure‐based drug design, Novartis developed the highly specific noncovalent Ω‐loop pocket inhibitor IAG933. This compound binds to the Ω‐loop pocket of TEAD through multiple hydrogen bonds and hydrophobic interactions, and directly competes with YAP binding [115]. A Phase 1 clinical trial (NCT04857372) was previously conducted. IAG933 demonstrates potent antiproliferative activity in NF2‐deficient mesothelioma cell lines, with half‐maximal growth inhibition (GI_50_) values ranging from 13 to 91 nM and half‐maximal inhibitory concentration (IC_50_) values for TEAD target gene inhibition between 11 and 26 nM [115]. It also shows robust activity in TAZ‐fusion‐positive cancer models, including NIH‐3T3 xenograft tumors stably expressing TAZ‐CAMTA1 or YAP‐MAML2 fusions, with IC_50_ values between 82 and 292 nM [115]. In vivo, oral administration of IAG933 achieves deep tumor regression in patient‐derived xenograft (PDX) models of malignant pleural mesothelioma (MPM), with responses observed in seven of nine PDX models, and induces complete tumor regression in the MPM (MSTO‐211H) orthotopic model at tolerated doses [115]. Notably, IAG933 exhibits synergistic activity with MAPK pathway inhibitors, including MEK inhibitors and KRAS‐selective inhibitors, in KRAS‐mutant pancreatic and lung cancer models, wherein combined targeting of MAPK and Hippo pathways induces synergistic lethality and more durable responses [115]. Specifically, the combination of IAG933 with the KRAS^G12C^ inhibitor JDQ443 demonstrates strong synergistic benefit in KRAS^G12C^‐mutated NSCLC and colorectal cancer (CRC) cell lines, and upfront addition of IAG933 deepens responses to JDQ443 in NCI‐H2122 xenografts [115]. These findings establish IAG933 as a first‐in‐class direct YAP/TAZ–TEAD PPI disrupter with broad applicability across Hippo‐driven and RAS‐MAPK‐altered malignancies. In Phase 1, IAG933 showed an objective response rate (ORR) of only 19% (37 patients) and 13% in the pleural mesothelioma subset (*n* = 30). Dose‑limiting toxicities included QTc prolongation and proteinuria [116]. This agent has since been discontinued by Novartis in November 2025. Targeting the Ω‐loop presents a core tension. While the approach achieves exquisite on‐target specificity, it delivers only incomplete blockade of the YAP–TEAD interface, a deficit that argues for combination strategies to achieve comprehensive pathway suppression.

### 4.2 TEAD PBP Inhibitors

These inhibitors bind to the PBP of TEAD, blocking autopalmitoylation, thereby reducing TEAD stability and transcriptional activity (Figure 2B). This is currently the fastest‐advancing class of inhibitors with the largest number of clinical and preclinical candidates. PBP inhibitors are typically classified into covalent irreversible inhibitors and noncovalent reversible inhibitors based on their mode of binding, while a third emerging category, subtype‐selective PBP inhibitors, exploits subtle structural differences between TEAD1–4 paralogs to achieve selectivity.

#### 4.2.1 Discovery and Mechanism Overview

The PBP was established as a druggable site with the discovery of LM98, a flufenamic acid analog showing strong affinity to TEAD, inhibition of autopalmitoylation, and reduction of YAP–TEAD transcriptional activity. The binding of LM98 to TEAD was supported by X‐ray crystallography, providing the first direct structural validation of PBP druggability [117]. Several complementary discovery strategies have since been employed to identify PBP‐binding chemotypes. Structure‐based virtual screening has yielded promising lead compounds. Hit compound L06 was identified as a potent TEAD4 inhibitor through docking‐based virtual screening. L06 inhibits TEAD autopalmitoylation, interrupts the YAP–TEAD interaction, and reduces YAP–TEAD transcriptional activity, with efficacy demonstrated in HCT‐116 CRC models [118]. JM7 was identified through structure‐based virtual ligand screening combined with biochemical and cell biological studies. JM7 inhibits YAP transcriptional reporter activity with an IC_50_ of 972 nM, inhibits TEAD palmitoylation rendering TEAD unstable, and directly binds TEAD1–4 in cells as confirmed by cellular thermal shift assay. JM7 significantly impairs proliferation, colony formation, and migration of mesothelioma (NCI‐H226), breast (MDA‐MB‐231), and ovarian (OVCAR‐8) cancer cells [119]. Covalent fragment screening has enabled the development of irreversible inhibitors. Covalent fragment screening followed by structure‐based design was employed to develop the irreversible TEAD inhibitor MYF‐03‐69. Covalent binding within the TEAD palmitate pocket disrupts YAP–TEAD association, suppresses TEAD transcriptional activity, and inhibits cell growth of Hippo signaling‐defective MPM. Cell viability screening across 903 cancer cell lines demonstrated a high correlation between YAP–TEAD dependency and sensitivity to MYF‐03‐69, providing the most comprehensive preclinical sensitivity profile for any TEAD inhibitor to date [103]. Fragment‐based discovery was extended to acrylamide chemotypes; cocrystal structures revealed binding to the conserved palmitoylation Cys of TEAD2/TEAD3 as well as to a nonconserved cysteine in TEAD2 closer to the YAP–TEAD interface, providing a structural basis for subtype‐selective covalent inhibitor design [120]. Phenotypic screening has also proven fruitful. A 5‐azaindole hit was obtained through phenotypic screening, and subsequent structural optimization yielded derivatives with diverse TEAD selectivity profiles, including pan‐TEAD inhibitors, TEAD3‐sparing inhibitors, and TEAD2‐selective atropisomers. These compounds demonstrated good single‐agent antitumor activity in lung cancer xenograft models [121].

#### 4.2.2 Covalent Irreversible Inhibitors

Covalent PBP inhibitors form a covalent bond with a conserved cysteine residue at the PBP, achieving sustained target engagement and high cellular potency. This class includes the most advanced clinical candidates in the YAP–TEAD field.

Structural evolution of covalent warheads. The electrophilic reactivity of the warhead is the primary determinant of both target‐engagement durability and off‐target toxicity. First‐generation covalent inhibitors such as K‑975 employ a chloroacrylamide warhead, which forms a stable thioether bond with the PBP cysteine but also carries a risk of nonspecific labeling of other reactive cysteine‐containing proteins. The next‐generation agent BPI‑460372 replaces chlorine with fluorine (2‑fluoro‑acrylamide); the reduced electrophilicity of the α‑fluoro‑acrylamide improves metabolic stability and markedly lowers off‐target covalent binding. In clinical pharmacokinetic analyses, BPI‑460372 demonstrated linear kinetics over a 10–80 mg dose range without the renal toxicity observed with some earlier warheads, suggesting that the toxicity profile can be decoupled from target engagement through fine‐tuning of warhead electronics. An orthogonal approach uses a vinyl sulfone warhead (CPD10/CPD13), which also irreversibly modifies the PBP cysteine but generates a distinct covalent adduct; these compounds exhibit synergistic activity with EGFR and KRAS inhibitors. Together, the structure activity relationship (SAR) landscape indicates that covalent engagement is not monolithic: incremental changes in warhead geometry and electron‐withdrawing character can rebalance potency, selectivity, and metabolic stability.

Mechanistically, the governing SAR principle is the tuning of intrinsic warhead electrophilicity so as to reconcile three competing requirements. A highly reactive warhead (e.g., chloroacrylamide) maximizes the rate of covalent bond formation (*k*_inact_/*K*_I_) and therefore target occupancy, but at the cost of indiscriminate labeling of off‐target glutathione and reactive‐cysteinome proteins, accelerated glutathione‐mediated clearance and a shortened metabolic half‐life. Attenuating electrophilicity slows the intrinsic reaction rate and favors a “bind‐first, react‐later” mechanism, in which reversible recognition of the palmitate pocket precedes covalent capture. This can be accomplished either by substituting the α‑chlorine with the less electron‑withdrawing fluorine (giving 2‑fluoroacrylamide) or by employing geometrically different Michael acceptors, including vinyl sulfones, vinyl sulfonamides, and simple acrylamides. Consequently, residence time and cellular potency become governed by noncovalent affinity rather than by raw chemical reactivity, widening the therapeutic window through lower off‐target thiol adduction, reduced idiosyncratic toxicity, and improved pharmacokinetic stability while preserving durable on‐target engagement. Vinylsulfonamide warheads exemplify this balance, as tunable α‐substituents permit graded reactivity while retaining hydrolytic stability. Acrylamide‐based binders, conversely, offer synthetic tractability but require careful electronic deactivation to avoid promiscuous cysteine labeling [122, 123]. Warhead selection is therefore not merely a potency decision but a determinant of selectivity, safety, and developability [124].

##### 4.2.2.1 K‐975

K‐975, developed by Kyowa Kirin, was among the first reported TEAD palmitoylation inhibitors [125]. Crystallographic analysis revealed that this chloroacrylamide‐containing compound forms a covalent bond with Cys359 of TEAD1 and the equivalent cysteine residues in TEAD2‐4, thereby irreversibly blocking palmitate binding to the palmitate‐binding pocket [125]. By covalently and irreversibly inhibiting TEAD autopalmitoylation, a process essential for its transcriptional activity, K‐975 demonstrates selectivity for TEAD over other palmitoylated proteins [125]. The covalent mechanism ensures prolonged target engagement, with TEAD inhibition persisting for over 24 h even after the compound is washed out [125]. K‐975 exhibits potent antitumor activity, particularly in the treatment of MPM, where it significantly inhibits tumor growth in xenograft models [125].

##### 4.2.2.2 BPI‐460372

BPI‐460372 is a next‐generation covalent inhibitor developed by Betta Pharmaceuticals, featuring a unique 2‐fluoro‐acrylamide warhead that confers enhanced metabolic stability compared to first‐generation acrylamide‐containing agents [126]. This compound exhibits potent inhibition of all TEAD1/2/3/4 isoforms and demonstrates higher selectivity with minimal off‐target covalent binding affinity [126]. In vitro and in vivo drug metabolism analyses have demonstrated that BPI‐460372 is primarily metabolized by cytochrome P450 enzymes CYP2D6, CYP3A4 and CYP1A2, with low clearance in human, monkey, and rat hepatocytes, indicating a favorable pharmacokinetic profile. A Phase 1 trial (NCT05789602) initiated in 2023 employs a dose‐escalation design with expansion cohorts in biomarker‐selected populations. Preliminary data indicate a favorable safety profile, with primarily hematologic adverse events, including thrombocytopenia and neutropenia, that are reversible and manageable with dose modification. Specially, it has demonstrated no evidence of the renal toxicities associated with certain other TEAD inhibitors and preliminary data indicate a favorable safety profile with linear pharmacokinetics over a 10–80 mg dose range supporting once‐daily dosing. No drug‐related grade ≥ 3 adverse events were reported, and early efficacy signals include disease stabilization and partial responses in heavily pretreated MPM patients [126, 127].

##### 4.2.2.3 MYF‐03‐69

MYF‐03‐69, developed through covalent fragment screening followed by structure‐based design, established the broadest preclinical sensitivity profile for any TEAD inhibitor to date by screening cell viability across 903 cancer cell lines, revealing a high correlation between YAP–TEAD dependency and drug sensitivity. Transcription profiling of MYF‐03‐69‐treated mesothelioma cells identified upregulation of proapoptotic signals (including BIM and BMF) and downregulation of YAP/TEAD target genes (*CTGF*, *CYR61*). MYF‐03‐69 inhibited the growth of MPM in vivo with a favorable therapeutic window and remains an important reference compound for mechanistic and pharmacological studies of pan‐TEAD covalent inhibitors [103].

##### 4.2.2.4 MRK‐A

MRK‐A is an aryl ether YAP1/TEAD inhibitor that demonstrated potent and specific inhibition of YAP1/TEAD activity, favorable tolerability and tumor regression in a mesothelioma CDX model. However, rapid resistance was observed; RNA‐seq of resistant tumors revealed marked upregulation of hepatocyte growth factor (HGF) as a key resistance mechanism [128].

##### 4.2.2.5 Next‐Generation Covalent Warheads

CPD10 and CPD13 represented a novel class of pan‐TEAD inhibitors that use a vinyl sulfone warhead to covalently bind the conserved cysteine at the entrance of the PBP while disrupting YAP–TEAD interactions. CPD10 and CPD13 exhibit nanomolar IC_50_ values against gastric cancer cells with Hippo pathway abnormalities and show synergistic killing in EGFR‐ and KRAS‐mutant NSCLC cells, suggesting potential to overcome resistance to existing targeted therapies. They are currently in preclinical optimization [129]. A covalent acrylamide fragment series was identified by fragment screening; cocrystal structures showed binding to the palmitoylation Cys of TEAD2 and TEAD3 as well as to a nonconserved cysteine in TEAD2 located closer to the YAP–TEAD interface, providing a structural foundation for subtype‐selective covalent inhibitor development [120].

#### 4.2.3 Noncovalent Reversible Inhibitors

To overcome the potential off‐target toxicity and long‐term safety concerns of covalent inhibitors, multiple research teams have developed noncovalent inhibitors targeting the PBP. These compounds bind through hydrophobic interactions and hydrogen bonds, enabling reversible inhibition of TEAD palmitoylation with potentially improved safety and a clearer path to subtype selectivity.

##### 4.2.3.1 VT3989

VT3989, developed by Vivace Therapeutics, is a first‐in‐class orally bioavailable TEAD palmitoylation inhibitor that targets the conserved PBP of TEAD transcription factors [130]. This compound exhibits potent biochemical activity against all TEAD paralogs (TEAD1–4) and demonstrates robust antitumor efficacy in MPM xenograft models, with dose‐dependent tumor regressions observed at well‐tolerated doses [131]. In the ongoing Phase 1/2 trial (NCT04665206), initiated in 2021, 172 patients have been enrolled, including 135 with mesothelioma. Among 22 MPM patients treated with optimized dosing schedules (50 or 100 mg, 2 weeks on/2 weeks off), the ORR was 32% with a disease control rate (DCR) of 86%. Several patients have achieved durable partial responses lasting beyond 18 months, with one ongoing response extending beyond 21 months, prompting advancement toward Phase 3 registration trials in 2026 [130, 132]. Adverse events were manageable with intermittent dosing schedules (including 2 weeks on/2 weeks off, 1 week on/3 weeks off and weekly dosing), which effectively mitigated albuminuria while maintaining efficacy [130]. No dose‐limiting cardiotoxicity was observed [133].

##### 4.2.3.2 IK‐930

IK‐930, developed by Ikena Oncology, is another reversible pan‐TEAD inhibitor that received FDA Fast Track designation in 2022 based on compelling preclinical data demonstrating potent inhibition of TEAD palmitoylation and YAP/TAZ‐driven transcription, with nanomolar potency in NF2‐mutant mesothelioma cell lines [134]. The Phase 1 study (NCT05228015) was designed to evaluate IK‐930 in NF2‐altered solid tumors, with particular emphasis on MPM and meningioma [135]. Ikena's development strategy initially emphasized biomarker‐driven patient selection and combination approaches with targeted agents and immunotherapy. However, in May 2024, Ikena Oncology announced the discontinuation of the IK‐930 program following an interim analysis that showed a 0% ORR in 14 evaluable patients. This disappointing outcome underscores the challenges of translating preclinical efficacy to clinical benefit and raises important questions about patient selection criteria and the potential need for prolonged target occupancy that reversible inhibitors may not achieve at tolerated doses.

##### 4.2.3.3 GNE‐7883

GNE‐7883, developed by Genentech, is a highly potent and selective pan‐TEAD inhibitor developed by Genentech, which binds to the TEAD PBP with high affinity, disrupting YAP/TAZ‐mediated transcriptional program [136]. It exhibits superior antiproliferative activity compared to early TEAD inhibitors in a broad panel of Hippo‐dysregulated tumor models, including mesothelioma, NF2‐mutant schwannomas, and CRC. Notably, GNE‐7883 has been demonstrated to overcome both intrinsic and acquired resistance to KRAS^G12C^ inhibitors in preclinical models by abrogating YAP‐driven bypass signaling, highlighting its potential in combination with RAS pathway inhibitors for KRAS‐mutant cancers [137].

#### 4.2.4 Subtype‐Selective Strategies

TEAD1–4 share greater than 70% sequence identity in the PBP, but subtle structural differences in pocket dimensions and adjacent surface features can be exploited to achieve subtype selectivity, a property of growing therapeutic interest given the distinct tissue distributions and physiological roles of TEAD paralogs.

##### 4.2.4.1 MSC‐1254 and MSC‐5046: Dual‐Mode TEAD1‐Selective Inhibitors

Remarkably, both a reversible inhibitor (MSC‐1254) and a covalent inhibitor (MSC‐5046) with TEAD1 selectivity were developed from the same chemical scaffold through structure‐based drug design. The TEAD1 preference was rationalized by steric differences in the lower pocket of the palmitoylation site between subtypes, with TEAD1 having the largest available volume to accommodate substitution in this region. This work demonstrates that subtype selectivity can be engineered from a common scaffold and that the mode of inhibition (reversible vs. covalent) can be tuned independently, providing important insights for the rational design of next‐generation TEAD inhibitors with tailored pharmacological properties [138].

##### 4.2.4.2 5‐Azaindole Atropisomers

Subtype selectivity was extended through phenotypic screening followed by structural optimization of a 5‐azaindole hit. The optimized series yielded derivatives with diverse TEAD selectivity profiles, including pan‐TEAD inhibitors, TEAD3‐sparing inhibitors, and TEAD2‐selective atropisomers. These compounds demonstrated good single‐agent antitumor activity in lung cancer xenograft models, illustrating that atropisomerism can be leveraged for subtype tuning [121].

### 4.3 TEAD Protein Degraders

Targeted protein degradation technologies, including PROTACs and molecular glues, provide an alternative therapeutic modality to occupancy‐based inhibition [139]. Compared with traditional inhibitors, degraders offer several advantages. Complete elimination of TEAD protein blocks all of its functions, including noncanonical activities such as transcriptional repression and cofactor‐independent scaffolding. Catalytic action allows effective degradation at substoichiometric concentrations, thereby reducing pharmacokinetic demands and enabling lower dosing regimens. Potential for tissue‐selective degradation exists through the tissue‐specific expression patterns of E3 ligases (Figure 2B).

#### 4.3.1 PROTAC Degraders

PROTAC molecules are heterobifunctional compounds composed of a TEAD ligand, an E3 ubiquitin ligase ligand and a connecting linker. By simultaneously engaging TEAD and an E3 ligase, PROTACs induce TEAD ubiquitination and proteasomal degradation.

##### 4.3.1.1 Compound 40 (H122)

Compound 40 (H122) is a CRBN ligand‐based pan‐TEAD PROTAC constructed by conjugating a TEAD covalent inhibitor to lenalidomide via a linker. This degrader exhibits picomolar to nanomolar half‐maximal degradation concentration (DC_50_) values against TEAD1 and, importantly, demonstrates robust antitumor efficacy in a mouse xenograft model derived from MSTO‐211H cells, providing the first in vivo validation of a TEAD‐targeting PROTAC. Mechanistic studies confirmed that H122‐induced degradation is dependent on CRBN engagement, E3 ligase activity and proteasome function, and that it effectively downregulates YAP target genes including *CTGF* and *CYR61* [140].

##### 4.3.1.2 HC278

HC278 selectively degrades TEAD1 and TEAD3 at low nanomolar concentrations while exhibiting minimal activity against TEAD2 and TEAD4. By engineering a stable cell line coexpressing all four epitope tagged TEAD paralogs, the investigators demonstrated that this selectivity arises from the preferential formation of stable ternary complexes with TEAD1 and TEAD3 in conjunction with CRBN and DNA binding protein 1 (DDB1), as confirmed by amplified luminescent proximity homogeneous assay (AlphaLISA) and global proteomic analyses. HC278 showed good antitumor activity in NF2‐mutant tumor models [139].

##### 4.3.1.3 Compound 27

Compound 27 is a PROTAC that achieves TEAD2‐selective degradation despite being derived from the pan‐TEAD inhibitor VT107, underscoring the critical role of linker optimization in dictating isoform specificity [141]. This compound was developed by linking a VT107 analog to a thalidomide ligand through an optimized linker, and systematic evaluation of antiproliferative effects against NF2‐deficient NCI‐H226 cells identified compound 27 as the lead molecule. Notably, compound 27 significantly reduced the transcription of YAP target genes including *CYR61* and *CTGF*, demonstrating functional engagement of the Hippo pathway. These findings exemplify how linker engineering can redirect the degradation profile of PROTACs derived from pan‐TEAD ligands toward specific paralogs, offering a novel strategy for the development of isoform‐selective TEAD degraders [141].

##### 4.3.1.4 KG‐FP‐003: A Potent TEAD PROTAC Degrader With Durable Activity

KG‐FP‐003 is a potent and selective TEAD‐targeting PROTAC degrader that achieves robust degradation at low nanomolar concentrations, with durable degradation kinetics and broad antitumor activity across multiple cancer types [142]. KG‐FP‐003 exemplifies the therapeutic potential of degrader approaches, with efficient degradation of all TEAD isoforms in a ubiquitin–proteasome system (UPS)‐dependent manner and robust therapeutic responses both in vitro and in vivo. These findings establish KG‐FP‐003 as a compelling lead candidate for isoform‐selective TEAD therapy and reveal its therapeutic promise beyond Hippo‐dysregulated mesothelioma [142].

#### 4.3.2 Molecular Glue Degraders/Modulators

Molecular glues represent an additional emerging strategy. Small molecules that induce novel interactions between YAP/TAZ and E3 ligases, or promote TEAD engagement with transcriptional repressors, could achieve inhibition through neomorphic mechanisms. Although still in early stages of discovery, such approaches may ultimately overcome the limitations of current small‐molecule inhibitors.

##### 4.3.2.1 Amine 1: First‐in‐Class TEAD Molecular Glue Degraders

Amphista Therapeutics has developed the first bona fide TEAD molecular glue degraders, representing a novel shift from conventional PROTAC architectures [143]. These compounds, exemplified by the optimized lead amine 1, feature a molecular weight of only approximately 655 Da, representing a significant reduction compared to traditional PROTAC degraders which typically exceed 800 Da. The amine‐based degrader scaffolds undergo extracellular conversion to reactive aldehyde species, which mediate covalent engagement of the E3 ligase F‐box protein 22 (FBXO22) Cys326, triggering TEAD proteasomal degradation. This research highlighted strategies for the SAR and rational optimization of aldehyde‐mediated degrons to generate novel precision molecular glue degraders against TEAD, a high value oncology target [143].

##### 4.3.2.2 VGLL4‐Recruiting Molecular Glue Modulators

A groundbreaking discovery showed that select sulfonamide‐containing TEAD‐targeting compounds act as molecular glues toward the repressive VGLL4–TEAD interaction [107]. These compounds enhance the interaction between TEAD and the transcriptional repressor VGLL4, and the chemically induced VGLL4–TEAD complexes confer an antiproliferative effect by outcompeting YAP–TEAD complexes at chromatin. This cofactor switch from YAP to VGLL4 reshapes transcriptional networks, including genes involved in cellular proliferation and mechanosignaling. VGLL4 overexpression confers sensitivity to these compounds in Hippo‐driven cell lines, and genetic deletion of VGLL4 completely abolishes cellular responsiveness to these molecules both in vitro and in vivo. These findings reveal a class of TEAD modulators with a fundamentally different mechanism, promoting transcriptional repression rather than simply disrupting activation [107]. These findings reveal a strategy for suppressing oncogenic Hippo pathway dysregulation in cancer and identify glue‐like molecules that enforce transcriptional repression.

##### 4.3.2.3 PITX2 as an Alternative YAP Partner Under TEAD Inhibition

A complementary finding revealed an unexpected consequence of TEAD inhibition [144]. In external auditory canal squamous cell carcinoma (EACSCC), the small‐molecule TEAD inhibitor VT104 not only inhibited the YAP–TEAD interaction but also induced YAP binding to paired‐like homeodomain transcription factor 2 (PITX2), suggesting that PITX2 represents an alternative partner transcription factor of YAP under TEAD‐inhibited conditions. Knockdown of PITX2 enhanced sensitivity to VT104, inhibiting cell growth and migration, whereas overexpression of PITX2 induced oncogenic gene expression programs as well as YAP/TEAD target genes, promoting tumor growth in vivo. Nuclear YAP and PITX2 were coexpressed in primary tumor tissues and significantly correlated with poor prognosis [144]. This finding indicates that the YAP cofactor landscape is more dynamic than previously appreciated, with implications for both resistance mechanisms and combination therapy strategies.

### 4.4 Inhibitors Targeting TEAD‐Cofactor Interactions

TEAD interacts with multiple non‐YAP cofactors that contribute to context‐specific transcriptional programs. Targeting these interactions represents a promising strategy for tissue‐ or pathway‐specific intervention.

#### 4.4.1 TEAD–FXR in Liver Cancer

The YAP–TEAD complex was discovered to directly bind FXR through a unique region of the TEAD YBD domain and to recruit HDAC1, thereby inhibiting FXR transcriptional activity and promoting liver tumorigenesis (Figure 2B). This interaction interface does not overlap with the YAP‐binding site. Based on this mechanism, researchers demonstrated that blocking repressor activity of YAP either by enhancing FXR function, inhibiting HDAC1, or promoting bile acid excretion through bile salt export pump (BSEP) upregulation reduces liver damage and cancer progression in experimental models, showing good antitumor activity with low toxicity to normal tissues [105].

#### 4.4.2 TEAD–VGLL1 in Endocrine‐Resistant Breast Cancer

Selective estrogen receptor degraders such as fulvestrant were found to promote expression of VGLL1, a coactivator for TEAD transcription factors, which acts via TEADs to drive growth of fulvestrant‐resistant breast cancer cells [145]. Pharmacological disruption of the VGLL1–TEAD4 interaction with verteporfin prevented the growth of resistant cells, and VGLL1‐directed EGFR upregulation sensitized fulvestrant‐resistant breast cancer cells to EGFR inhibitors [145]. Chromatin immunoprecipitation followed by sequencing (ChIP‐seq) analysis revealed that VGLL1 is recruited to TEAD4 binding regions in fulvestrant‐resistant cells, with cobinding at the EGFR gene locus driving its transcriptional activation [145]. This work supports VGLL1–TEAD4 as a tractable cofactor‐interaction node in endocrine‐resistant disease and is further discussed in the context of acquired resistance mechanisms (Figure 2B).

**FIGURE 3:**
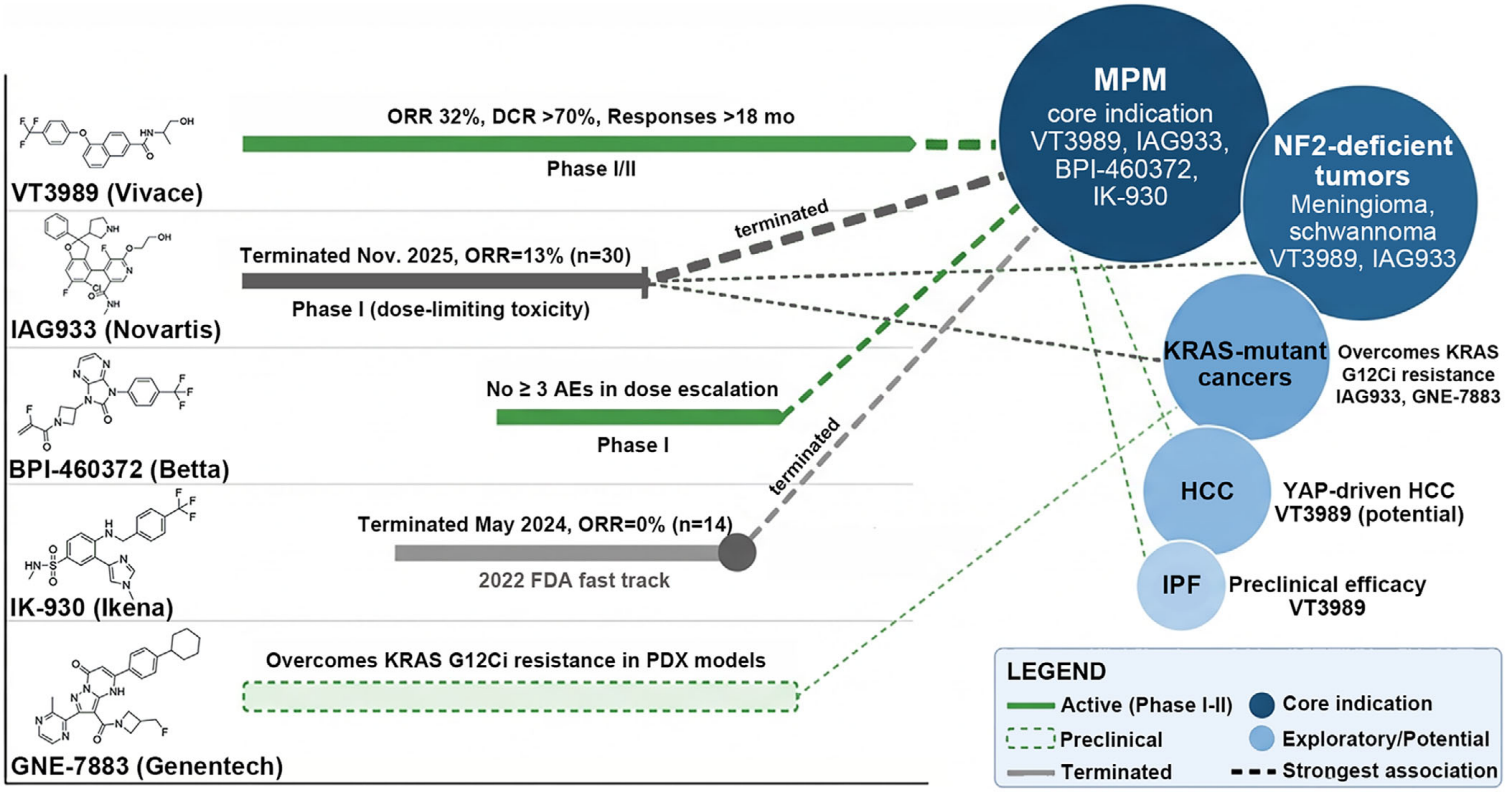
Clinical development landscape and indication layout of TEAD inhibitors. (Left) Clinical development timeline. VT3989 (Vivace) is in Phase 1/2 that achieved an ORR of 32% and a DCR of 86% in patients with NF2‑mutant MPM, with some responses lasting more than 18 months. IAG933 (Novartis) entered Phase 1 and showed synergy with MEK inhibitors in KRAS‑mutant models, but has since been discontinued owing to insufficient clinical efficacy. BPI‑460372 (Betta) is in Phase 1, with parallel clinical studies in China and the United States demonstrating linear pharmacokinetics and no drug‑related adverse events of grade ≥ 3. IK‑930 (Ikena) advanced to Phase 1 but was discontinued in May 2024 after an interim analysis showed an ORR of 0% among 14 evaluable patients. GNE‑7883 (Genentech) is in preclinical development that has been shown to overcome acquired resistance to sotorasib in KRAS^G12C^‑mutant non–small‐cell lung cancer PDX models. (Right) Indication bubble chart. The major indications for TEAD inhibitors are displayed, with bubble size reflecting the level of research activity or clinical evidence strength. MPM represents the core indication, with all active drugs represented and the most prominent efficacy data associated with VT3989. NF2‑deficient solid tumors (including meningioma, schwannoma, and renal cell carcinoma) represent an important expansion direction for VT3989 and IAG933. KRAS‑mutant cancers (non–small‐cell lung cancer, pancreatic cancer) are key exploratory areas for IAG933 and GNE‑7883, where combination with MEK or KRAS^G12C^ inhibitors may overcome adaptive resistance. HCC and IPF are potential indications supported by preclinical studies showing therapeutic benefit of TEAD inhibition. The connecting lines illustrate the correspondence between each drug and the indications, with line thickness reflecting the strength of the association.

Together, these examples illustrate the broader concept that TEAD is a node in a network of context‐specific cofactor interactions, each potentially druggable for tissue‐ or disease‐selective intervention. A comprehensive overview of the TEAD‐targeting agent landscape and the corresponding indications is provided in Figure 3, with preclinical agents summarized in Table 1 and clinical‐stage agents in Table 2.

**TABLE 1:** Preclinical therapies targeting the Hippo–YAP/TAZ–TEAD pathway.

Compound	Mechanism	Binding site	Stage	Key indications	Status
Patent‐22	YAP–TEAD PPI disruptor; used to validate a fluorescence‐polarization assay at the YAP Ω‐loop binding region	Ω‐loop pocket	Tool compound/assay reference	Hippo‐driven tumors [112]	Preclinical tool compound
LM98	Flufenamic acid analogue; binds TEAD, inhibits autopalmitoylation and reduces YAP–TEAD transcriptional activity	PBP	Hit/structural validation	Hippo‐altered tumors [117]	Preclinical; first X‐ray validation of PBP druggability
L06	TEAD4 inhibitor from docking‐based virtual screening; blocks autopalmitoylation and interrupts the YAP–TEAD interaction	PBP	Hit	Colorectal cancer (HCT‐116) [118]	Preclinical
JM7	Structure‐based virtual ligand screening hit; inhibits palmitoylation and destabilizes TEAD; binds TEAD1–4 in cells (CETSA)	PBP	Hit‐to‐lead	Mesothelioma (NCI‐H226), breast (MDA‐MB‐231), ovarian (OVCAR‐8) [119]	Preclinical
5‐Azaindole atropisomers	Phenotypic‐screening hit optimized into pan‐TEAD, TEAD3‐sparing and TEAD2‐selective atropisomers	PBP	Hit‐to‐lead; subtype‐selective series	Lung cancer xenografts [121]	Preclinical; atropisomerism used as a subtype‐tuning handle
K‐975	Covalent irreversible pan‐TEAD inhibitor; chloroacrylamide warhead bonds Cys359 of TEAD1 and the equivalent cysteines in TEAD2–4	PBP (conserved Cys)	Advanced preclinical	MPM; combinations with cisplatin/pemetrexed, palbociclib and osimertinib [125]	Preclinical
MYF‐03‐69	Covalent irreversible pan‐TEAD inhibitor from covalent fragment screening followed by structure‐based design	PBP (conserved Cys)	Advanced preclinical/reference compound	Hippo‐defective MPM [103]	Preclinical; broadest sensitivity map to date (903 cancer cell lines)
MRK‐A	Aryl‐ether YAP1/TEAD inhibitor; potent and specific inhibition of YAP1/TEAD activity	PBP	Preclinical	Mesothelioma (CDX model) [128]	Preclinical; tumor regression but rapid resistance via HGF upregulation
CPD10/CPD13	Covalent pan‐TEAD inhibitors bearing a vinyl sulfone warhead that generates a distinct covalent adduct at the cysteine at the PBP entrance	PBP entrance Cys	Preclinical optimization	Gastric cancer with Hippo abnormalities; synergy in EGFR‐ and KRAS‐mutant NSCLC [129]	Preclinical optimization
Acrylamide covalent fragments	Fragment‐derived covalent binders of the palmitoylation cysteine of TEAD2/TEAD3 and of a nonconserved TEAD2 cysteine closer to the YAP–TEAD interface	PBP + adjacent nonconserved Cys (TEAD2)	Fragment/structural series	Structural basis for subtype‐selective covalent design [120]	Preclinical
GNE‐7883	Highly potent and selective allosteric noncovalent pan‐TEAD inhibitor disrupting YAP/TAZ‐mediated transcription	PBP (allosteric)	Advanced preclinical	MPM, NF2‐mutant schwannoma, colorectal cancer; overcomes intrinsic and acquired KRAS^G12C^‐inhibitor resistance [136, 137]	Preclinical; resistance via AP‐1/FOSL1 is reversed by MAPK inhibitors
VT104 (VT1)/VT2	Noncovalent pan‐TEAD autopalmitoylation inhibitors	PBP	Preclinical	NF2‐null schwannoma (vestibular and dorsal root ganglion); external auditory canal SCC	Preclinical; VT104 induces YAP–PITX2 cofactor escape [144]
VT107	Noncovalent pan‐TEAD inhibitor	PBP	Preclinical; chemical starting point for degraders	NF2‐deficient tumors	Preclinical; parent ligand of the TEAD2‐selective PROTAC compound 27 [141]
MSC‐1254	Reversible TEAD1‐selective inhibitor; selectivity rationalized by the larger available lower‐pocket volume of TEAD1	PBP (lower pocket)	Preclinical	TEAD1‐selective proof‐of‐concept [138]	Preclinical
MSC‐5046	Covalent TEAD1‐selective inhibitor built on the same scaffold as MSC‐1254	PBP (lower pocket)	Preclinical	TEAD1‐selective proof‐of‐concept [138]	Preclinical; shows mode of inhibition can be tuned independently of paralog selectivity
Compound 40 (H122)	CRBN‐based pan‐TEAD PROTAC (covalent TEAD ligand conjugated to lenalidomide)	PBP ligand + CRBN	Preclinical, in vivo validated	MPM (MSTO‐211H xenograft) [140]	Preclinical; first in vivo validation of a TEAD‐targeting PROTAC
HC278	CRBN/DDB1 PROTAC degrading TEAD1 and TEAD3 at low nanomolar concentrations while sparing TEAD2/TEAD4, via preferential ternary‐complex formation	PBP ligand + CRBN	Preclinical	NF2‐mutant tumors; TEAD4‐selective degraders proposed for pulmonary fibrosis [139]	Preclinical
Compound 27	TEAD2‐selective PROTAC (VT107 analogue linked to thalidomide); linker optimization redirects paralog specificity from a pan‐TEAD ligand	PBP ligand + CRBN	Preclinical	NF2‐deficient NCI‐H226 [141]	Preclinical
KG‐FP‐003	Pan‐TEAD PROTAC degrader; potent, ubiquitin‐proteasome‐system‐dependent degrader of all TEAD isoforms with durable kinetics	PBP ligand + E3 ligase	Preclinical lead	Broad antitumor activity extending beyond Hippo‐dysregulated mesothelioma [142]	Preclinical lead candidate
Amine 1	Molecular glue degrader; extracellular conversion to a reactive aldehyde covalently engages FBXO22 Cys326 to trigger TEAD proteasomal degradation	TEAD + FBXO22	Preclinical	TEAD‐dependent oncology [143]	Preclinical; first bona fide TEAD molecular glue degrader
XMU‐MP‐1	Reversible MST1/2 inhibitor; transiently activates YAP to promote tissue repair	MST1/2 kinase	Advanced preclinical	Liver and intestinal repair and regeneration [78]	Preclinical
TRULI (Lats‐IN‐1)	ATP‐competitive LATS1/2 inhibitor; reversibly activates YAP	LATS1/2 kinase	Tool compound	Postmitotic‐tissue regeneration (inner ear, heart, retina) [79]	Preclinical
Super‐TDU	VGLL4‐mimetic peptide; competitively blocks YAP–TEAD binding	TEAD YBD (YAP interface)	Preclinical	Gastric cancer [147]	Preclinical; peptide/gene‐deliverable modality
YAP bioPROTAC	Anti‐YAP nanobody fused to the RNF4 RING domain; degrades endogenous YAP; AAV/LNP‐deliverable	YAP + RNF4 E3 ligase	Preclinical, in vivo validated	YAP‐dependent cancers [148]	Preclinical; first gene‐encoded YAP degrader
YAP siRNA formulations	RNAi knockdown of YAP via LNP, polymeric nanococktail or aptamer‐directed nanovesicle	YAP1 mRNA	Preclinical	HCC; EGFR‐TKI‐resistant NSCLC; cholangiocarcinoma [149, 150, 151]	Preclinical

**TABLE 2:** Representative TEAD‐targeting agents that have entered human clinical trials.

Compound	Mechanism and binding site	TEAD paralog selectivity	Clinical trial (NCT number) and phase	Biomarker/patient‐selection strategy	Efficacy	Key adverse events	Development status
VT3989	Reversible, noncovalent PBP inhibitor	Pan‐TEAD	NCT04665206, Phase 1/2	NF2 alteration / Merlin loss; Merlin–YAP dual‐label IHC developed as a response‐predictive assay [155]	MPM: ORR ∼32%, DCR 86% with optimized intermittent dosing; durable partial responses > 18–21 months [130, 131, 132, 133]	Proteinuria/albuminuria (reversible, mitigated by intermittent schedules), peripheral edema, fatigue; no dose‐limiting cardiotoxicity	Ongoing; advancing to Phase 3 registration trials in 2026
IK‐930	Reversible PBP inhibitor	Pan‐TEAD	NCT05228015, Phase 1	NF2‐altered solid tumors, with emphasis on MPM and meningioma	ORR 0% in 14 evaluable patients [134, 135]	Tolerable safety profile; limited exposure owing to lack of efficacy	Discontinued (May 2024)
BPI‐460372	Covalent PBP inhibitor (2‐fluoro‐acrylamide warhead)	Pan‐TEAD	NCT05789602, Phase 1	Biomarker‐selected expansion cohorts (NF2‐mutant tumors; MPM; meningioma)	Disease stabilization and partial responses in heavily pretreated MPM [126, 127]	Reversible hematologic AEs (thrombocytopenia, neutropenia); no drug‐related grade ≥ 3 AEs; no renal toxicity to date	Ongoing
IAG933	Direct YAP–TEAD PPI inhibitor (Ω‐loop pocket)	Pan‐TEAD	NCT04857372, Phase 1	NF2‐deficient MPM; YAP/TAZ‐fusion‐positive tumors; combinations in KRAS‐ and EGFR‐mutant cancers	ORR 19% overall (*n* = 37); 13% in the pleural mesothelioma subset (*n* = 30) [115, 116]	Dose‐limiting QTc prolongation and proteinuria	Discontinued (November 2025)

*Note*: The table representatively summarizes the four agents with publicly disclosed clinical data. More than 15 agents have entered clinical development globally, of which approximately 12 remain in active development.

### 4.5 Targeting Upstream Hippo Pathway Kinases: MST1/2 and LATS1/2 Modulators

Although current drug development has concentrated on the downstream TEAD node, the upstream core kinases MST1/2 and LATS1/2 constitute mechanistically attractive intervention points, because restoring their activity would reimpose pathway control irrespective of TEAD pocket mutations or cofactor switching. Direct pharmacological activation of a kinase is, however, intrinsically more difficult than inhibition, and no direct MST1/2 or LATS1/2 agonist has yet entered clinical development. Pathway activation can instead be achieved by disabling negative regulators of the core cassette. The MARK2/3 kinases, which directly phosphorylate NF2 and YAP/TAZ and thereby reverse LATS1/2‐mediated tumor suppression, represent the first druggable “agonist‐by‐proxy” route. Genetic or protein‐based MARK2/3 blockade restored Hippo pathway activity and regressed established YAP/TAZ‐dependent tumors in vivo [86].

Conversely, transient inhibition of MST1/2 or LATS1/2 is a validated strategy for regenerative indications. The reversible MST1/2 inhibitor XMU‐MP‐1 augments intestinal repair and liver regeneration in both acute and chronic injury models and enhances repopulation of human hepatocytes in vivo [78]. The ATP‐competitive LATS inhibitor TRULI reversibly activates YAP and promotes proliferation of postmitotic supporting cells, CMs, and Müller glia [79], and the optimized LATS inhibitor NIBR–LTSi expands tissue stem cells and accelerates liver regeneration after extended hepatectomy [146]. Pharmacological LATS1/2 inhibition likewise enhances periodontal tissue regeneration [80] and corneal endothelial regeneration [81]. The clinical development of upstream kinase modulators therefore faces a fundamental bidirectional challenge. Chronic pathway activation is required for oncology, whereas transient, tissue‐restricted inhibition is required for regeneration. The dual requirement necessitates targeted delivery and a tightly controlled dosing window.

### 4.6 Direct YAP/TAZ Inhibitors and YAP/TAZ Degraders

All clinical‐stage agents to date target TEAD, leaving the YAP/TAZ effectors themselves pharmacologically unexploited. Direct targeting of YAP/TAZ is attractive because it would neutralize both TEAD‐dependent and TEAD‐independent functions and permit functional discrimination between the two paralogs. Nevertheless, the approach is chemically formidable, as YAP and TAZ are largely intrinsically disordered and lack well‐defined ligandable pockets. Several strategies are nonetheless emerging. Peptide‐based inhibitors that mimic natural TEAD‐binding partners can disrupt the complex from the effector side. For instance, the VGLL4‐mimetic peptide Super‐TDU potently suppresses YAP‐driven gastric tumor growth in preclinical models [147], and stapled or cyclic YAP Ω‐loop‐mimetic peptides recapitulate the highest‐affinity interaction interface. More recently, targeted protein degradation has been extended to YAP itself. A nanobody‐based bioPROTAC, generated by fusing a high‐affinity anti‐YAP nanobody to the RING domain of the E3 ligase RNF4, selectively degrades endogenous YAP through the UPS and inhibits the progression of YAP‐dependent tumors in vitro and in vivo; critically, the degrader can be delivered as a gene by lipid nanoparticles or adeno‐associated virus (AAV), establishing proof‐of‐concept for gene‐encoded YAP degraders [148]. Small‐molecule YAP/TAZ degraders remain at an earlier stage, but the validation of β‐TrCP‐ and RNF4‐mediated ubiquitination as endogenous YAP/TAZ disposal routes suggests that heterobifunctional recruiters of these ligases could achieve small‐molecule YAP/TAZ degradation, circumventing the resistance liabilities of TEAD pocket occupancy.

### 4.7 Gene Therapy, RNA‐Based Therapeutics, and Antibody‐ and Cell‐Based Modalities

Targeting the Hippo–YAP/TAZ–TEAD pathway extends beyond small‐molecule inhibitors to biologic and genetic modalities, several of which have yielded compelling preclinical proof‐of‐concept evidence. RNA‐based therapeutics are the most advanced. Lipid‐nanoparticle‐formulated YAP siRNA restored hepatocyte differentiation and caused pronounced tumor regression in a genetically engineered hepatocellular carcinoma (HCC) model [149], and a stimuli‐responsive dendritic‐polymer nanococktail codelivering gefitinib and YAP siRNA overcame EGFR‐TKI resistance in NSCLC xenograft and patient‐derived models [150]. Tumor‐targeted delivery has been further refined with aptamer‐directed milk‐derived nanovesicles carrying YAP siRNA, which suppressed cholangiocarcinoma growth and synergized with chemotherapy in preclinical models [151], and with peptide‐modified lipid nanoparticles that improve tumor‐selective delivery of RNA therapeutics [152]. Antisense oligonucleotides against YAP1 or TAZ represent a chemically distinct and delivery‐friendly alternative that remains largely unexplored.

Gene‐, antibody‐, and cell‐based therapies are likewise emerging. AAV‐ or nanoparticle‐mediated delivery of the nanobody‐bioPROTAC gene achieved intratumoral YAP degradation in vivo [148], and delivery of VGLL4‐mimetic constructs such as Super‐TDU provides a genetic means of reimposing transcriptional repression on TEAD [147]. Antibody‐derived modalities are represented by the high‐affinity anti‐YAP nanobodies underlying the bioPROTAC strategy [148], which could in principle be formatted as intracellular intrabodies or fusion biologics. In the cell‐therapy arena, YAP functions as an immunosuppressive brake on T‐cell activation and tumor infiltration. Consequently, YAP inhibition in T cells enhances their antitumor activity [153], supporting the incorporation of YAP/TAZ modulation into CAR‐T engineering. In addition, mechanobiological control of YAP/TAZ‐mediated mechanosensing is increasingly recognized as a lever to improve the efficacy and manufacturability of engineered T‐cell therapies [154]. Collectively, these modalities broaden the therapeutic landscape from occupancy‐based small‐molecule inhibition toward programmable, tissue‐restricted pathway control. However, all of these approaches remain at preclinical or conceptual stages and face delivery, durability and safety hurdles distinct from those of small molecules.

Collectively, these modalities differ in their structural targets, resistance and toxicity profiles, and developability. Noncovalent reversible PBP inhibitors and paralog‐selective degraders currently offer the most attractive balance of potency, mutation resilience and manageable safety. In contrast, direct PPI disruptors and cofactor‐interface modulators remain earlier in validation (Table 3).

**TABLE 3:** Comparison of YAP–TEAD‐targeting modalities.

Modality	Mechanism	Advantages	Disadvantages	Resistance liabilities	Toxicity concerns	Clinical maturity	Best‐fit indications
PPI inhibitors	Competitively occupy the Ω‐loop surface pocket to block the YAP/TAZ–TEAD interface	Do not require the palmitate pocket; independent of Cys mutation; pan‐TEAD blockade of transcription	Shallow, largely polar interface makes high‐affinity, drug‐like binding difficult; oral PK historically limiting	Interface point mutations; cofactor (VGLL) switching restoring transcription	QTc prolongation and proteinuria were the dose‐limiting toxicities in Phase 1	Phase 1 completed but the lead agent (IAG933) has been discontinued	NF2‐deficient MPM; TAZ/YAP‐fusion tumors; combination with KRAS or EGFR inhibitors in RAS‐MAPK‐altered disease [83, 112, 113, 114, 115, 116, 144]
Covalent PBP inhibitors	Irreversibly modify the conserved palmitate‐pocket cysteine (chloro‐/fluoro‐acrylamide, vinyl sulfone)	High, durable target occupancy; strong cellular potency; prolonged pharmacodynamic effect	Off‐target thiol labeling; idiosyncratic toxicity risk; ineffective against pocket Cys mutation	Palmitate‐pocket cysteine mutation (e.g., C359S); MAPK/AP‐1 bypass	Renal (albuminuria/proteinuria) and GI toxicity; warhead‐dependent off‐target effects	Phase 1 (BPI‐460372) plus several mature preclinical assets	NF2‐deficient MPM where prolonged occupancy is required; combination with EGFR‐TKIs or KRAS inhibitors to eradicate drug‐tolerant persister cells [103, 120, 122, 123, 124, 125, 126, 127, 128, 129]
Noncovalent PBP inhibitors	Reversibly occupy the palmitate pocket via hydrophobic/H‐bond contacts (e.g., VT3989, GNE‐7883)	Retain activity against Cys mutants; tunable selectivity; reversible engagement may widen safety margin	Require sustained exposure; potency driven by affinity; possible efflux susceptibility	Alternative Cys‐independent resistance; VGLL cofactor switching; drug efflux	Reversible, schedule‐manageable renal/GI toxicity; intermittent dosing mitigates	The most clinically advanced modality, VT3989 is progressing to Phase 3	NF2‐altered MPM and solid tumors; the preferred mechanism for chronic nononcology use such as fibrosis, where reversibility protects regeneration [117, 118, 119, 130, 131, 132, 133, 134, 135, 136, 137]
PROTAC degraders	Heterobifunctional recruitment of an E3 ligase to catalytically degrade TEAD (e.g., HC278, KG‐FP‐003)	Event‐driven, catalytic; remove scaffolding functions; overcome Cys mutation and cofactor resistance	Large MW/poor oral PK; ternary‐complex optimization; E3 ligase‐dependent resistance	E3 ligase downregulation; VGLL switching less relevant if TEAD removed	Deeper pathway suppression may narrow therapeutic window; paralog‐selective degraders mitigate	Preclinical; in vivo validation achieved (compound 40/H122) but no clinical‐stage agent	Tumors resistant to occupancy‐based inhibitors and settings requiring complete pathway ablation; TEAD4‐selective degraders for pulmonary fibrosis while sparing cardiac TEAD1 [139, 140, 141, 142]
Molecular glues	Monovalent compounds that reshape TEAD surfaces to induce degradation or recruit repressive cofactors (e.g., amine 1, VGLL4‐mimetics)	Small, drug‐like; catalytic degradation; can convert TEAD into a repressive complex	Mechanism dependent on cofactor context; efficacy tied to VGLL4 status	Loss of VGLL4 expression abolishes glue‐dependent repression	Deeper pathway suppression may narrow the therapeutic window; VGLL4 dependence may yield unpredictable tissue effects; no clinical safety data	Early preclinical	Hippo‐driven tumors with documented high VGLL4 (and, where relevant, VGLL3) expression, which should be a prerequisite for trial entry rather than a retrospective correlate [107, 143]
Cofactor modulators	Target TEAD–cofactor (e.g., VGLL, AP‐1) surfaces for tissue/disease‐selective intervention	Potential tissue‐ or disease‐selective effects; spare housekeeping TEAD functions	Least clinically mature; context‐specific; validation limited	Redundant cofactor usage; incomplete pathway blockade	Potentially narrower toxicity due to selectivity; unproven	Earliest stage, proof‐of‐concept and tool compounds only	Liver cancer via the TEAD–FXR/HDAC1 axis; endocrine‐resistant breast cancer via VGLL1–TEAD4, where disruption also resensitizes cells to EGFR inhibitors [105, 107, 144, 145]
Upstream kinase modulators	Inhibit MST1/2 or LATS1/2 to transiently activate YAP/TAZ (regeneration), or restore their tumor‐suppressive activity via MARK2/3 or STRIPAK blockade (oncology) [78, 79, 86]	Orthogonal to TEAD pocket mutations and cofactor switching; enables pharmacological regeneration	Direct kinase activation is pharmacologically difficult; bidirectional dosing challenge (activation vs. inhibition)	Not yet defined	Systemic YAP/TAZ activation risks tumorigenesis; requires transient, tissue‐restricted dosing	Preclinical (XMU‐MP‐1, TRULI, NIBR–LTSi) [78, 79, 146]	Regenerative medicine (liver, intestine, periodontal, cornea); MARK2/3 inhibition in YAP/TAZ‐dependent cancer [80, 81, 86, 156]
Gene‐, RNA‐, antibody‐, and cell‐based modalities	siRNA/ASO knockdown of YAP1/WWTR1; AAV‐ or LNP‐delivered nanobody bioPROTAC; VGLL4‐mimetic constructs; YAP/TAZ‐modulated engineered T cells [147, 148, 152, 154]	Target YAP directly; programmable and tissue‐restricted; gene‐encoded degraders possible	Delivery, durability and immunogenicity hurdles	Largely undefined	Delivery‐ and vector‐related toxicity; immunogenicity	Early preclinical proof‐of‐concept [148, 149, 151]	HCC and cholangiocarcinoma (siRNA), EGFR‐TKI‐resistant NSCLC, YAP‐dependent tumors; engineered T‐cell therapies [149, 150, 151, 154, 157]

## 5 Preclinical and Clinical Research Progress

Building upon a deepened understanding of the YAP–TEAD interaction and groundbreaking medicinal chemistry optimization, multiple TEAD inhibitors have advanced into clinical development. In this section, we critically appraise their performance across oncology, fibrosis, immunology, and regenerative medicine, building on the drug‐centric and mechanism‐modality comparisons summarized in Tables 1, 2, 3.

### 5.1 Oncology Applications

Research on YAP–TEAD inhibitors in cancer therapy is the most advanced, with more than 15 agents have entered clinical development globally, of which approximately 12 remain in active development, mainly targeting NF2‐mutant tumors and YAP‐overexpressing solid tumors.

#### 5.1.1 NF2‐Mutant Tumors

NF2 mutations result in loss of Merlin protein function, which relieves inhibition of the Hippo pathway, leading to sustained YAP/TAZ activation. These tumors are the most sensitive to TEAD inhibitors. MPM is characterized by NF2 mutations in approximately 70% of cases. The landmark Phase 1 data for VT3989 have established the first clinical proof‐of‐concept for TEAD‐targeted therapy in genetically selected populations, as detailed in Section 4.2.3. BPI‐460372 is also being evaluated in NF2‐mutant tumors with an emphasis on biomarker‐selected expansion cohorts, and preliminary efficacy signals have been reported [126, 127].

Meningioma represents another NF2‐mutant tumor type, with approximately 60% of sporadic meningiomas harboring NF2 or other Hippo pathway gene mutations. Preliminary clinical data with BPI‐460372 in this population are awaited. Furthermore, schwannomas and ependymomas present additional opportunities, as these nervous system tumors frequently arise in patients with germline NF2 mutations. While surgical resection remains standard of care, TEAD inhibitors may offer systemic therapy for patients with multiple bilateral vestibular schwannomas or spinal ependymomas, for whom surgery carries high morbidity. Preclinical studies utilizing the TEAD autopalmitoylation inhibitors VT1 (VT104) and VT2 in NF2‐null mouse models of schwannoma demonstrated significant reductions in vestibular ganglion and dorsal root ganglion tumor volumes following 21 days of treatment, accompanied by increased apoptosis observed in tumor cells. These pan‐TEAD inhibitors also blocked proliferation in primary human schwannoma cells at nanomolar concentrations and significantly reduced tumor size in vivo [158].

#### 5.1.2 Other Solid Tumors

For solid tumors with wild‐type Hippo pathway but YAP overexpression, TEAD inhibitors have limited efficacy as monotherapy, but show good potential in combination with other targeted agents or immunotherapy. In KRAS‐mutant NSCLC, preclinical studies show that TEAD inhibitors in combination with KRAS^G12C^ inhibitors overcome resistance, with the allosteric pan‐TEAD inhibitor GNE‐7883 enhancing sotorasib response and achieving dramatic tumor regression in treatment‐naive and resistant PDX models [136]. In EGFR‐mutant NSCLC, short‐term EGFR‐TKI treatment induces YAP activation through NF2 loss and nuclear YAP1/WWTR1 accumulation, which represents an important mechanism of acquired resistance to EGFR‐TKIs [159]. Pharmacological coinhibition of YAP or TEAD or genetic deletion of YAP1, depletes dormant persister cells by enhancing EGFR/MEK‐induced apoptosis [160]. Preclinical studies show that combining the TEAD inhibitor K‐975 with osimertinib almost completely ablates persister cancer cells compared to osimertinib alone, and combining MYF‐01‐37 with osimertinib reverses osimertinib resistance in NF2‐deleted PC‐9, HCC827 and HCC4006 cells [161]. In human epidermal growth factor receptor 2 (HER2)‐positive breast cancer, Enhancer of zeste homolog 2 (EZH2) inhibitors sensitize HER2‐positive tumors to HER2 kinase inhibitors through epigenetic reprogramming, with a mechanism involving cooperative effects on YAP and proapoptotic regulators. Specifically, EZH2 normally silences Bcl‐2 modifying factor (BMF) by methylating H3K27 at the BMF locus, and EZH2 inhibitors promote H3K27me3 release, but this stimulates binding of repressive YAP/TEAD complexes which still restrict BMF expression. However, in the presence of EZH2 inhibitors, HER2 kinase inhibitors trigger dissociation of repressive YAP/TEAD complexes, potently upregulate BMF, and induce apoptosis in resistant cells. Accordingly, EZH2 inhibitors cooperate with genetic or pharmacologic inhibition of YAP/TEAD, which similarly induces BMF expression and triggers apoptosis, providing a new therapeutic strategy for HER2‐positive resistant breast cancer [162].

### 5.2 Applications in Fibrotic Diseases

**FIGURE 4:**
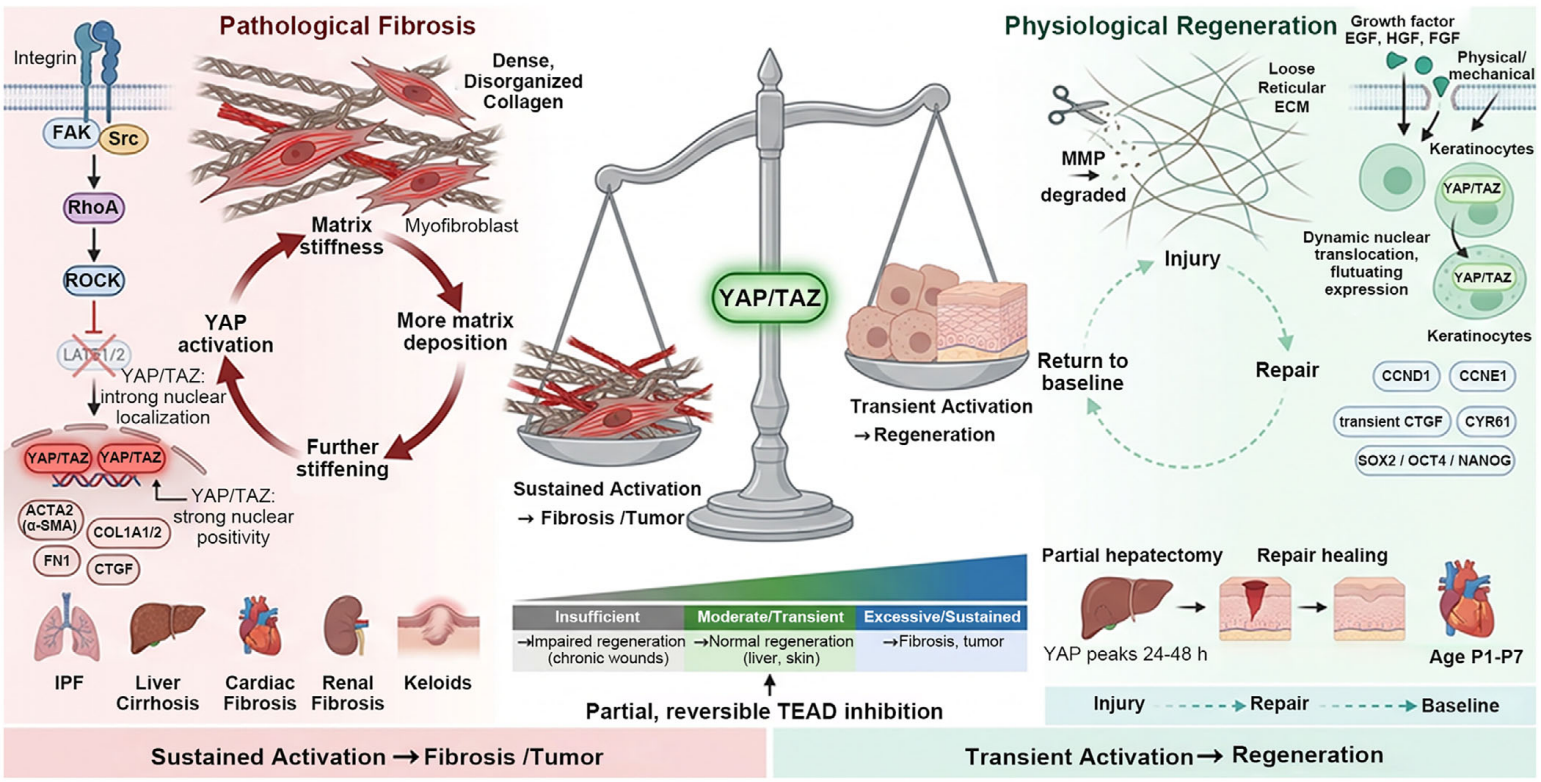
Dual role of the Hippo‐YAP/TAZ pathway in fibrosis and regeneration. (Left) Pathological fibrosis. Under conditions of stiff ECM, TGF‑β stimulation, and sustained mechanical stress, YAP/TAZ is constitutively activated (nuclear localization) in fibroblasts, driving high expression of profibrotic genes such as α‑SMA, COL1A1/2, FN1, and CTGF. This promotes the transition of fibroblasts into myofibroblasts, excessive ECM deposition and ultimately the formation of irreversible fibrotic scars, as seen in IPF, liver fibrosis, cardiac fibrosis, renal fibrosis, and keloids. Thick red arrows denote sustained activation and a positive‑feedback vicious cycle (matrix stiffening → YAP activation → further matrix deposition). (Center) YAP/TAZ as a regeneration‐fibrosis balance hub. Insufficient YAP/TAZ activity impairs regeneration (chronic wounds); moderate (transient) activity supports normal regeneration (liver regeneration, skin healing); excessive (sustained) activity drives fibrosis, pathological hyperplasia, and tumorigenesis (ADPKD, cardiac hypertrophy, fibrosis, cancer). Therefore, partial and reversible TEAD inhibition holds promise for achieving antifibrotic/anticancer efficacy while preserving tissue regenerative capacity. (Right) Physiological regeneration. Following tissue injury (partial hepatectomy, skin wounding), YAP/TAZ is transiently activated in hepatocytes, keratinocytes, or stem cells, driving expression of proproliferative genes including CCND1 and CCNE1, thereby promoting cell proliferation and tissue repair. After repair is completed, YAP/TAZ activity returns to baseline levels. Thin green dashed arrows indicate transient activation and a self‑limiting process.

Beyond oncology, the Hippo–YAP/TAZ pathway plays a central role in fibrogenesis across multiple organ systems, positioning TEAD inhibitors as potential therapeutics for fibrotic diseases. A summary of the contrasting roles of YAP/TAZ in fibrosis and regeneration is depicted in Figure 4, highlighting the therapeutic window for TEAD inhibition.

IPF represents the most lethal interstitial lung disease. Despite the availability of approved antifibrotic therapies, including nintedanib and pirfenidone, median survival remains only 3–5 years [163]. Genetic or pharmacological inhibition of YAP attenuates bleomycin‐induced pulmonary fibrosis in mice [164]. Targeted deletion of YAP/TAZ in lung fibroblasts significantly dampens fibroinflammatory responses, decreases myofibroblast activation, and attenuates fibrosis while concurrently enhancing alveolar epithelial cell regeneration following bleomycin injury [164]. Notably, verteporfin has been shown to not only prevent but also reverse established pulmonary fibrosis by downregulating TIMP levels and enhancing MMP1 and MMP9 activation to promote Collagen I degradation, an ability that significantly surpasses current antifibrotic agents which merely slow disease progression [164]. Pharmacological inhibition of YAP–TEAD interaction using verteporfin has been shown to suppress mechanical stress‐induced fibrotic ECM remodeling in nucleus pulposus cells, reducing the expression of Collagen I and α‐SMA [165]. This cross‐tissue mechanism conservation supports TEAD inhibitors as a broad therapeutic strategy for fibrotic diseases.

Chronic liver injury from viral hepatitis, alcohol consumption, or NASH drives progressive fibrosis that culminates in cirrhosis and HCC. HSCs, the primary collagen‐producing cells in the liver, exhibit marked YAP activation during transdifferentiation into myofibroblasts [166]. Conditional deletion of YAP in HSCs prevents experimental liver fibrosis, whereas postinjury YAP deletion promotes fibrosis resolution [167]. Specifically, selective YAP depletion in myofibroblast HSCs induces senescence and confers protection against liver fibrosis in both bile duct ligation and CCl_4_‐induced injury models, although these senescent cells concurrently secrete senescence‐associated secretory phenotype factors that may perpetuate low‐grade inflammation, suggesting that combined senolytic strategies could optimize antifibrotic outcomes [167, 168]. The liver presents unique opportunities and challenges for TEAD inhibitor therapy. On one hand, the high prevalence of YAP‐driven HCC creates potential for dual antifibrotic and anticancer efficacy. Verteporfin inhibits YAP‐induced hepatomegaly and tumorigenesis, and has been shown to reverse YAP‐induced sorafenib resistance in HCC models by enhancing sorafenib‐induced apoptosis and suppressing tumor growth [169]. On the other hand, the essential role of YAP in hepatocyte proliferation and liver regeneration raises concerns regarding impaired wound healing. YAP is pivotal in regulating liver growth and regeneration, and pharmacological inhibition of YAP–TEAD interaction using verteporfin significantly attenuates hepatomegaly and liver regeneration following partial hepatectomy by reducing Ki67‐positive hepatocyte numbers and downregulating vascular endothelial growth factor (VEGF) and HGF expression [170]. Preclinical studies suggest that the timing and context of YAP/TAZ inhibition are critical, with injury‐prior inhibition exacerbating damage while postinjury targeting of activated HSCs promoting resolution [171]. This temporal dependency has profound implications for clinical trial design, potentially necessitating HSC‐specific delivery systems or intermittent dosing regimens to preserve regenerative capacity while maximizing antifibrotic efficacy.

Myocardial fibrosis contributes to the pathogenesis of heart failure with preserved ejection fraction (HFpEF) and to adverse remodeling post‐MI. Cardiac fibroblasts exhibit YAP activation in response to mechanical stress and neurohormonal stimuli, driving ECM deposition and diastolic dysfunction [172]. However, TEAD1 is essential for CM survival and cardiac development, necessitating the development of paralog‐selective inhibitors that spare TEAD1 in CMs while targeting TEAD2/3/4 in fibroblasts [173]. TEAD1 is required for maintaining adult CM excitation–contraction coupling, mitochondrial function, and oxidative stress response through direct transcriptional regulation of sarco/endoplasmic reticulum calcium ATPase 2a (SERCA2a), inhibitor‐1 (I‐1) and nuclear factor erythroid 2‐related factor 2 (NRF2) [174, 175]. Notably, mosaic knockout studies demonstrate that TEAD1‐deficient CMs exhibit intrinsic oxidative stress even at baseline, with marked exacerbation following angiotensin II stimulation and elevated 8‐hydroxydeoxyguanosine staining indicating oxidative DNA damage, establishing a cell‐autonomous mechanism for TEAD1‐mediated cardioprotection [175]. These findings underscore the need for paralog‐selective inhibitors that spare TEAD1 in CMs while targeting TEAD2–4 in fibroblasts.

In chronic kidney disease, YAP activation in tubular epithelial cells and interstitial fibroblasts promotes EMT and fibrogenesis [62]. Elevated nuclear YAP staining has been observed in renal tubular epithelium during regeneration and fibrogenesis stages after acute kidney injury, as well as in the interstitium of patients with IgA nephropathy and membranous nephropathy [176]. YAP/TAZ inhibition has demonstrated renoprotective effects in preclinical models of diabetic nephropathy and unilateral ureteral obstruction [62]. Collectively, YAP/TAZ serve as central drivers in various organ fibrosis, yet they are equally indispensable for normal tissue regeneration. This dual nature presents unique challenges and opportunities for the clinical application of TEAD inhibitors.

### 5.3 Immunology Applications

Emerging evidence positions YAP/TAZ–TEAD as critical regulators of tumor‐immune interactions. While YAP and TAZ have complex, cell‐type‐specific roles in immune regulation, several nodes are directly actionable by TEAD inhibitors and hold immediate translational relevance. YAP and TAZ have opposing functions in T‐cell differentiation. In detail, YAP is highly expressed in immunosuppressive regulatory T cells (Tregs) and reinforces their functions, while TAZ inhibits Treg differentiation and promotes inflammatory Th17 cells [47, 74, 75]. In cluster of differentiation 8‐positive (CD8^+^) T cells, high YAP expression correlates with poor survival across cancers [153], as YAP inhibits cytotoxic differentiation and drives dysfunction. In the innate immune system, YAP promotes NLRP3 inflammasome activation [73] and modulates macrophage polarization through HDAC3–NCoR1‐mediated repression of arginase‐1 [71]. These divergent cell‐type‐specific roles create a “double‐edged sword” for systemic pan‐TEAD inhibition: while blocking YAP in tumor cells may alleviate immunosuppression and augment antitumor immunity, simultaneously suppressing YAP in effector T cells or disrupting dendritic cell maturation could impair antitumor immune responses [47]. This complexity necessitates cell‐selective targeting strategies, such as tumor‐specific TEAD blockade via prodrugs or antibody–drug conjugates (ADCs).

#### 5.3.1 Tumor‐Intrinsic Immune Evasion

YAP/TAZ promote tumor‐immune evasion through direct transcriptional regulation of immune checkpoints. The Hippo pathway induces expression of CD24, a “don't eat me” signal that inhibits macrophage phagocytosis by engaging Siglec‐10 [177]. CD24 is highly expressed in TNBC, ovarian cancer and B‐cell lymphomas, and its expression correlates with YAP activity and poor prognosis [177, 178]. Verteporfin and TEAD inhibitors suppress CD24 expression by blocking YAP–TEAD binding to the CD24 promoter, thereby sensitizing tumors to macrophage‐mediated clearance and providing a rationale for combination with macrophage checkpoint modulators [177]. Additionally, YAP/TAZ drive expression of programmed death ligand‐1 (PD‐L1) through direct TEAD binding to its promoter, establishing a mechanistic link between Hippo pathway activation and immune checkpoint expression [179]. TAZ directly interacts with the PD‐L1 promoter through TEAD family transcription factors, and deletion scanning of the PD‐L1 promoter has identified a putative TEAD‐responsive element [179]. In human MPM, YAP regulates PD‐L1 through a similar mechanism, transcriptionally modulating PD‐L1 via enhancer binding and inhibiting T‐cell function [180]. These findings suggest potential synergy between TEAD inhibitors and anti‐PD‐1/PD‐L1 antibodies.

#### 5.3.2 T‐Cell Considerations for Therapeutic Targeting

The dual effect of YAP/TAZ on T cells by promoting Tregs while suppressing effector CD8^+^ T‐cell function presents a therapeutic challenge. Systemic YAP/TAZ inhibition may impair CD8^+^ T‐cell function while simultaneously reducing Treg‐mediated suppression. Conditional deletion of YAP in T cells enhances antitumor immunity and improves response to immune checkpoint blockade in preclinical models [153], yet the net effect of pharmacological TEAD inhibition on the tumor‐immune microenvironment remains to be fully characterized in patients. Optimal strategies may require tumor cell‐specific TEAD inhibition, achieved through prodrug formulations or tumor‐targeted delivery, as well as careful scheduling to preserve antitumor immunity.

#### 5.3.3 Cancer‐Associated Fibroblasts

CAFs are abundant stromal cells that promote tumor progression through ECM remodeling, angiogenesis and immunosuppression. Single‐cell transcriptomics has revealed that myofibroblastic CAFs (myCAFs), the most protumorigenic subset, exhibit high YAP/TAZ activity driven by matrix stiffness and TGF‐β signaling [181]. Preclinical studies suggest that YAP/TAZ inhibition in CAFs may suppress their protumorigenic functions, potentially reducing collagen deposition and ECM remodeling [182]. This stromal targeting may partly account for the efficacy of TEAD inhibitors in desmoplastic tumors such as pancreatic ductal adenocarcinoma (PDAC), where CAFs constitute up to 90% of tumor mass [183]. However, recent studies have identified heterogeneity in CAF populations, with some subsets exhibiting tumor‐restraining functions. In PDAC, αSMA^+^ myCAFs have been shown to exert tumor‐restraining activity by mechanically restraining tumor expansion, while fibroblast activation protein‐positive (FAP^+^) CAFs are predominantly tumor promoting [184]. The differential impact of TEAD inhibition on these distinct CAF subsets requires further investigation, as indiscriminate suppression of all CAF populations may inadvertently compromise the tumor‐restraining mechanical barrier while targeting protumorigenic functions.

### 5.4 Regenerative Medicine and Stem‐Cell Biology

Unlike the sustained activation of YAP/TAZ observed in fibrosis and cancer, physiological regeneration requires transient activation of YAP/TAZ. Upon tissue injury (such as partial hepatectomy or cutaneous wounding), YAP/TAZ transiently translocate into the nucleus in stem or progenitor cells to orchestrate proliferation and repair, subsequently returning to baseline levels (Figure 4). Imbalance in this dynamic regulation, wherein insufficient activity impairs regeneration while excessive activity drives fibrosis or tumorigenesis, constitutes the theoretical foundation for the therapeutic window of TEAD inhibitors.

#### 5.4.1 Wound Healing and Tissue Repair

Nuclear localization of YAP and TAZ is necessary for skin wound healing, and their knockdown markedly delays the rate of wound closure [185]. Conditional knockout of YAP/TAZ in adult mouse epidermis impairs wound closure due to reduced epidermal cell proliferation, and loss of YAP in keratinocytes initiates premature terminal differentiation [186]. These findings raise concerns about impaired wound healing in patients receiving TEAD inhibitors, particularly those undergoing surgical procedures. Temporary discontinuation of therapy or local administration strategies may mitigate these risks. Conversely, excessive YAP activation underlies pathological scarring and keloid formation. In both keloids and hypertrophic scars, nuclear expression of YAP, TAZ, and TEAD is increased across all cell types, particularly in fibroblasts [187], suggesting that TEAD inhibitors may have therapeutic utility in preventing or treating pathological scarring.

#### 5.4.2 Regenerative Activation Strategies

Experimental activation of YAP/TAZ in mice promotes regeneration in organs with limited intrinsic regenerative capacity, such as adult heart and liver [188]. YAP agonists significantly promote muscle regeneration and skin wound healing in aged mice [23, 24]. The LATS inhibitor NIBR–LTSi demonstrates that pharmacological YAP activation can expand tissue stem cells and promote regeneration, including accelerating liver regeneration after extended hepatectomy in mice [146]. However, increased proliferation and cell dedifferentiation observed in heart, lung, kidney, and intestine preclude prolonged systemic LATS inhibition, underscoring the need for targeted delivery or local administration strategies [146]. Importantly, organ overgrowth induced by transient YAP overproduction is reversible upon cessation of activation, and YAP/TAZ activation alone is insufficient to drive cancer [189]. Therefore, the administration period of YAP/TAZ modulators should be strictly controlled to avoid tumor formation. Photo‐controlled or conditionally activatable small molecules may ultimately enable precise spatiotemporal regulation, though these technologies remain largely theoretical for YAP–TEAD modulators.

In summary, TEAD inhibitors have progressed from structure‐guided discovery to first‐in‐human testing, with VT3989 providing the clearest early clinical proof‐of‐concept in NF2‐deficient mesothelioma and encouraging preclinical rationale accumulating in fibrosis, cardiovascular disease and regenerative medicine. Nevertheless, the discontinuations of IAG933 and IK‐930 underscore that genetic selection alone is insufficient and that durable clinical benefit remains to be demonstrated.

## 6 Molecular Mechanisms of Resistance and Future Directions

**FIGURE 5:**
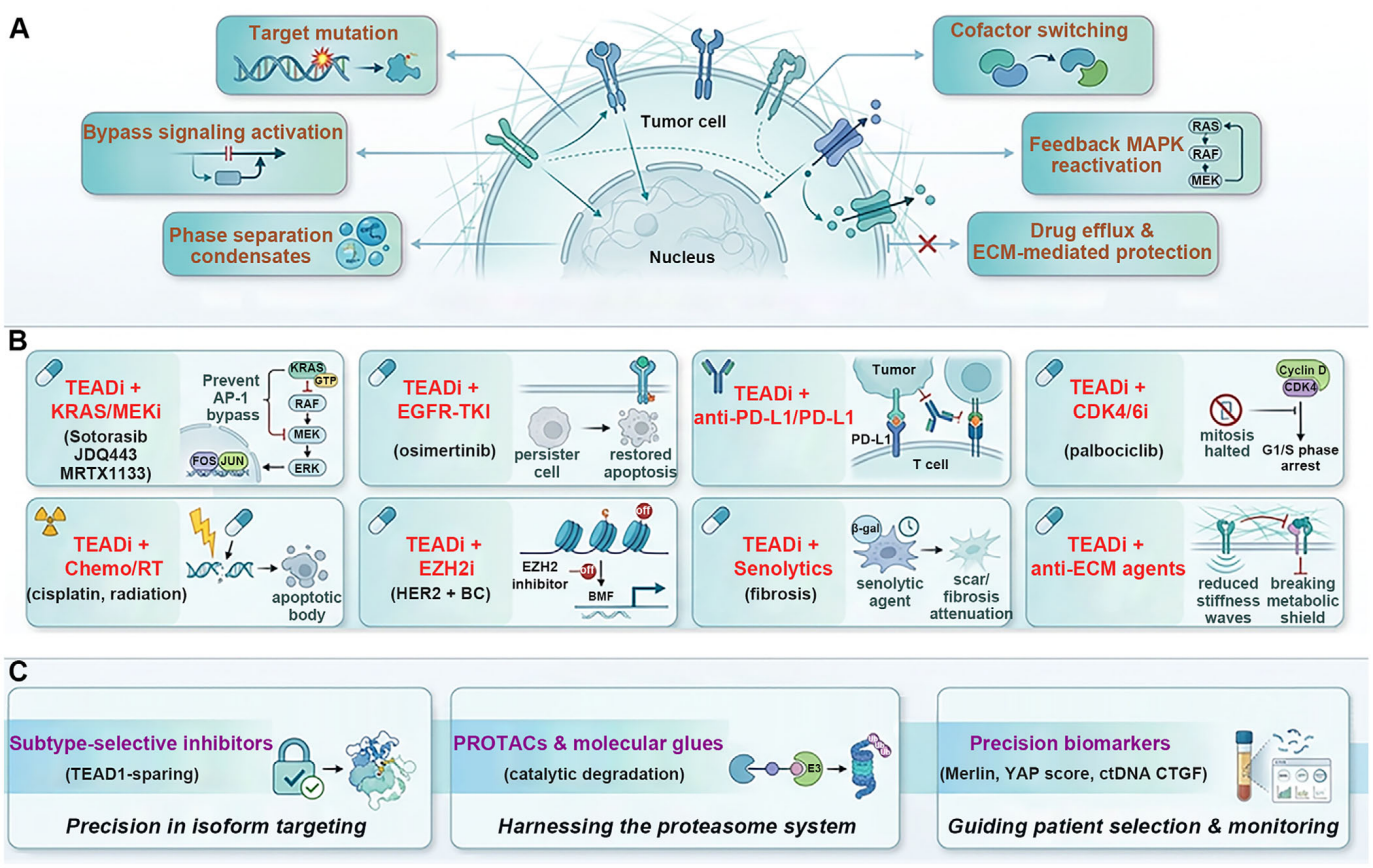
Mechanisms of resistance to TEAD inhibition and rational combination strategies for durable therapeutic responses. (A) Current challenges associated with TEAD inhibition. Multiple adaptive and intrinsic resistance mechanisms converge to attenuate therapeutic durability, including target mutation, cofactor switching, bypass signaling activation, feedback MAPK reactivation, phase‐separation condensate formation, and drug efflux/ECM‐mediated protection. These overlapping resistance programs collectively sustain oncogenic transcriptional signaling and tumor‐cell survival despite TEAD blockade. (B) Rational combination strategies designed to overcome distinct resistance. Representative approaches include: (i) TEADi combined with KRAS/MAPK inhibitors (sotorasib, JDQ443, MRTX1133) to suppress AP‐1/FOSL1‐mediated bypass signaling; (ii) TEADi plus EGFR‐TKIs (osimertinib) to eliminate drug‐tolerant persister (DTP) cells; (iii) TEADi with anti‐PD‐1/PD‐L1 immunotherapy to reverse immune evasion mediated by PD‐L1 and CD24; (iv) TEADi combined with CDK4/6 inhibitors (palbociclib) to restrain compensatory cell‐cycle progression; (v) TEADi plus chemotherapy or radiotherapy to enhance DNA damage and apoptosis; (vi) TEADi with EZH2 inhibition to restore proapoptotic transcriptional programs such as BMF derepression; (vii) TEADi combined with senolytic therapies to target YAP‐depleted senescent HSCs and attenuate fibrosis; and (viii) TEADi with anti‐ECM strategies to disrupt integrin/ROCK‐dependent stiffness signaling and metabolic protection. (C) Emerging future optimization strategies including subtype‐selective inhibitors with TEAD1‐sparing properties to reduce toxicity, PROTACs, and molecular glues for catalytic degradation of TEAD/YAP pathway components, and precision biomarker approaches (Merlin status, YAP transcriptional scores, and circulating ctDNA/*CTGF* markers) to guide patient stratification, treatment selection, and therapeutic monitoring.

Despite significant progress in the development of YAP–TEAD inhibitors, preclinical and early clinical studies have revealed that resistance is multifactorial and likely to limit the durability of single‐agent responses. In this section, we synthesize the mechanisms of intrinsic and acquired resistance, critically assess the unresolved challenges in the field, and propose a forward‐looking agenda to catalyze next‐generation drug development and clinical translation. The multifaceted nature of resistance to TEAD inhibitors, together with the rational design of combination therapies and emerging strategies to improve therapeutic durability, is summarized in Figure 5.

### 6.1 Molecular Mechanisms of Resistance to TEAD‐Targeted Therapy

Drug resistance to TEAD inhibitors encompasses both intrinsic and acquired mechanisms that frequently co‑occur in treated tumors. As illustrated in Figure 5, these include on‑target cysteine mutations, cofactor switching (VGLL4 loss with VGLL3 or PITX2 gain), bypass signaling via MAPK–AP‑1 (FOSL1) and TGFβ–SMAD cascades, feedback reactivation through LATS1/2 inactivation and Tet methylcytosine dioxygenase 1 (TET1)‐mediated TEAD demethylation, TEAD‑independent transcriptional condensates formed by LLPS of YAP, and microenvironmental protection conferred by drug efflux pumps and ECM stiffness.

#### 6.1.1 Intrinsic Resistance

##### 6.1.1.1 YAP/TAZ Functional Redundancy

YAP and TAZ share extensive target‐gene overlap; consequently, selective inhibition of YAP or TEAD can be offset by compensatory upregulation of TAZ. In YAP‐knockout tumor cells, TAZ mRNA and protein levels increase, sustaining oncogenic transcriptional output [177]. This redundancy necessitates pan‐YAP/TAZ targeting or complete TEAD family blockade for maximal pathway suppression.

##### 6.1.1.2 Bypass Pathway Activation

Hippo pathway target genes can be activated by alternative signaling cascades. TGFβ signaling directly regulates the expression of YAP target genes such as *CTGF* and *CYR61* through SMAD complexes, thereby cooperating with or bypassing YAP–TEAD inhibition [190]. This cross‐pathway crosstalk limits the single‐agent activity of TEAD inhibitors in TGFβ‐rich microenvironments.

##### 6.1.1.3 VGLL4 Expression Loss

The efficacy of molecular glue TEAD inhibitors critically depends on VGLL4 expression levels [107]. A subset of malignant mesotheliomas exhibits VGLL4 loss, rendering these tumors resistant to molecular glue inhibitors [107]. Preclinical studies demonstrated a positive correlation between VGLL4 expression level and sensitivity to molecular glue compounds, with high VGLL4‐expressing cells exhibiting significantly lower IC_50_ values compared to low‐expressing cells [107]. These findings establish VGLL4 as a predictive biomarker for this mechanistic class. Because loss of VGLL4 abolishes sensitivity to molecular glue and cofactor‐switching agents, we propose that documented high VGLL4 (and, where relevant, VGLL3) expression could be a rigorous prerequisite for patient inclusion in future trials of these degrader/glue modalities, rather than a retrospective correlative endpoint.

##### 6.1.1.4 Drug Efflux Pump Overexpression

Overexpression of drug efflux pumps such as P‐glycoprotein (P‐gp) and BCRP lowers intracellular TEAD inhibitor concentrations, contributing to intrinsic resistance [191]. This mechanism may be especially relevant for reversible inhibitors with shorter target residence times.

#### 6.1.2 Acquired Resistance

##### 6.1.2.1 Target Mutations

Mutations in TEAD proteins represent the most common acquired resistance mechanism. Mutations within the PBP can prevent covalent bond formation with the conserved cysteine residue or reduce inhibitor binding affinity by altering pocket geometry and introducing steric hindrance [192]. The spectrum of resistance‐conferring mutations likely differs between covalent and noncovalent PBP inhibitors; notably, cysteine mutations are specifically detrimental to covalent warhead engagement.

##### 6.1.2.2 Feedback Reactivation

Paradoxically, sustained YAP–TEAD inhibition can lead to compensatory upregulation of Hippo pathway components through alternative transcriptional mechanisms, potentially reactivating signaling and limiting therapeutic efficacy [193]. In addition, the YAP target TET1 physically interacts with TEAD‐bound genomic DNA, inducing localized DNA demethylation and transcriptional upregulation of TEAD1 and TEAD4, thereby creating a positive feedback loop that sustains oncogenic output [193]. Loss‐of‐function mutations in the upstream kinases LATS1/2 also drive resistance by preventing YAP/TAZ phosphorylation and promoting constitutive nuclear accumulation [194].

##### 6.1.2.3 VGLL3 Compensatory Activation

A subset of malignant mesotheliomas upregulates VGLL3 expression upon TEAD inhibitor treatment [195]. VGLL3 binds TEAD without inhibiting its transcriptional activity; instead, it drives the expression of oncogenic factors such as PIK3C2B and SOX4, enabling escape from inhibitor action [195]. This cofactor switching illustrates the dynamic remodeling of TEAD complex composition under therapeutic pressure.

##### 6.1.2.4 TEAD‐Independent YAP/TAZ Functions

Nuclear YAP can form liquid‐like condensates and regulate transcription independently of TEAD binding, sustaining cell survival in the presence of TEAD inhibitors [195]. YAP and TAZ also exert TEAD‐independent transcriptional functions by partnering with SMADs, β‐catenin and p73 [196]. Because all clinical‐stage inhibitors currently target TEAD, they cannot modulate these alternative interactions or discriminate between the protumorigenic and tumor‐suppressive functions of YAP/TAZ, potentially accounting for incomplete responses in some malignancies.

##### 6.1.2.5 Alternative Transcription Factor Engagement

Under TEAD inhibition, YAP can engage alternative transcription factors. The TEAD inhibitor VT104 was shown to induce YAP–PITX2 binding in EACSCC, activating an alternative oncogenic gene expression program that promoted tumor growth in vivo [144]. Coexpression of nuclear YAP and PITX2 significantly correlated with poor prognosis [144], suggesting that PITX2 expression may serve as a resistance biomarker.

##### 6.1.2.6 MAPK Pathway Feedback Activation

Enhanced AP‐1 transcription factor activity and restored YAP/TEAD chromatin occupancy jointly mediate resistance to the allosteric pan‐TEAD inhibitor GNE‐7883. MAPK inhibitors reduce FOSL1 expression and resensitize resistant cells both in vitro and in vivo [197]. These findings support early combination of TEAD inhibitors with MAPK inhibitors to delay the onset of resistance.

#### 6.1.3 Microenvironmental and Metabolic Adaptation

Even when TEAD is effectively inhibited, the tumor microenvironment can sustain residual YAP/TAZ activity. High ECM stiffness maintains YAP/TAZ nuclear localization through integrin–Rho/ROCK signaling independently of canonical Hippo signaling [198]. Additionally, resistant tumors can undergo metabolic rewiring such as upregulation of glutaminolysis reduces their dependence on YAP–TEAD‐driven metabolic gene programs [64]. These microenvironmental and metabolic adaptations act in concert with cell‐intrinsic resistance mechanisms to limit the durability of TEAD inhibitor responses.

### 6.2 Safety Liabilities and Therapeutic‐Window Challenges

Because YAP/TAZ–TEAD signaling governs tissue repair, cardiac contractile homeostasis, intestinal epithelial renewal, stem‐cell maintenance, and regenerative responses across multiple organ systems, systemic TEAD inhibition carries mechanism‐based, on‐target safety liabilities that extend well beyond the gastrointestinal and renal effects captured in early‐phase trials [130, 137, 199, 200]. Consequently, a rigorous translational appraisal therefore requires an organ‐by‐organ assessment of these liabilities and an explicit comparison of how they differ across pharmacological modalities. Specifically, cardiac toxicity emerges as the foremost concern, since TEAD1 is indispensable for CM survival and contractile function, and CM‐specific ablation of *Tead1* in adult mice produces a rapidly lethal dilated cardiomyopathy accompanied by mitochondrial dysfunction and necroptotic CM death [173, 201]. Pan‐TEAD inhibition, particularly by agents with substantial TEAD1 activity, therefore carries a tangible risk of cardiac dysfunction and heart failure. This consideration is the dominant driver for the development of TEAD1‐sparing, paralog‐selective scaffolds and for serial cardiac functional monitoring (echocardiography, troponin, NT‐proBNP) in clinical protocols. Additionally, renal toxicity represents a prominent on‐target liability, given that TEAD proteins are expressed in the renal proximal tubular epithelium. Pan‐TEAD inhibitors, including the noncovalent, reversible agent VT3989 and covalent members of the class, have produced dose‐dependent albuminuria and proteinuria that behave as a class effect [130]. This liability is, however, at least partly schedule‐ and scaffold‐dependent. Intermittent dosing markedly reduces albuminuria, and the covalent, irreversible TEAD1/3/4 inhibitor BPI‐460372 has so far shown a manageable renal profile in early clinical and preclinical datasets, although longer follow‐up is required [126]. The available GNE‐7883 allosteric inhibitor likewise avoids the proximal‐tubule signature observed with some covalent agents [136], supporting the view that warhead chemistry and lipophilicity shape this toxicity. Furthermore, intestinal and epithelial toxicity is largely on‐target and common to all modalities, because YAP/TAZ activity sustains intestinal stem‐cell renewal and epithelial barrier integrity [10], and inducible deletion of *Yap/Taz* in adult visceral smooth muscle causes a rapidly lethal colonic pseudo‐obstruction, underscoring the pathway's role in gut homeostasis [202]. Pharmacological TEAD inhibition impairs epithelial turnover and manifests clinically as nausea, diarrhea and mucosal injury. These effects are predominantly on‐target and most pronounced with pan‐TEAD agents, and they are mitigated mainly through intermittent scheduling, dose optimization, and supportive care rather than through structural modification. Moreover, impaired wound healing and regeneration represents another significant risk, since transient YAP/TAZ activation is required for re‐epithelialization, cutaneous wound repair and liver regeneration [15, 156, 203]. In portal‐vein‐ligation and partial‐hepatectomy models, YAP/TAZ signaling in activated HSCs drives hepatocyte hypertrophy and functional maturation, with downstream engagement of canonical targets [204, 205]. Sustained or degrader‐mediated suppression may therefore impair wound healing and blunt regenerative responses. This concern favors reversible inhibitors, deliberate treatment holidays, and the avoidance of perioperative dosing whenever clinically feasible. In addition, reproductive and developmental risks must be considered. Preclinical models have established essential roles for the YAP/TAZ–TEAD axis in early embryogenesis, foregut and cardiovascular development, and in adult tissue homeostasis [202, 206, 207]. Consequently, chronic TEAD inhibition carries a theoretical teratogenic and fertility risk. Adequate contraception, avoidance in pregnancy, and formal developmental and reproductive toxicology (DART) studies are warranted before broad or long‐term clinical use. For chronic‐dosing considerations in nononcology indications, the therapeutic window is narrowest in fibrotic and cardiovascular diseases, where prolonged administration in patients with long life expectancy amplifies the cumulative risk of the toxicities described above. In fibroblasts, YAP/TAZ are central regulators of ECM homeostasis and tissue fibrosis [63], and sustained pathway suppression could either blunt pathological fibrosis or, conversely, impair matrix turnover and repair. This context most strongly demands paralog‐ or tissue‐selective agents, local or targeted delivery, and intermittent dosing regimens. Finally, it is crucial to recognize that these liabilities are not uniform across drug classes, as modality‐dependent differences are pronounced. Pan‐TEAD agents such as GNE‐7883 [136] and the clinical‐stage VT3989 [130] carry the broadest on‐target risk, dominated by cardiac (TEAD1) and renal effects. Covalent inhibitors exemplified by K‐975 [125], the ethacrynic‐acid‐derived series [208], and BPI‐460372 [126] add warhead‐dependent off‐target and idiosyncratic toxicities, with IAG933, the first‐in‐class direct YAP–TEAD interface disruptor representing an alternative modality that achieves paralog‐wide engagement without occupying the palmitate pocket [115]. Noncovalent reversible inhibitors, including the recently described cobimetinib‐derived series [200], offer schedule‐manageable, potentially reversible toxicity. Paralog‐selective (TEAD1‐sparing) agents are specifically engineered to uncouple efficacy from cardiac risk. Degraders (PROTACs and molecular glues) elicit the deepest pathway suppression by ablating both scaffolding and transcriptional activities [137, 199, 209], which may further narrow the therapeutic window unless paralog selectivity is deliberately incorporated.

### 6.3 Future Directions

Three overarching priorities have emerged to guide the next generation of TEAD‑targeted therapies (Figure 5): (1) the development of subtype‑selective inhibitors that spare TEAD1 in the heart while targeting TEAD2–4 in fibrosis and cancer; (2) the advancement of PROTACs and molecular glues that catalytically degrade TEAD, thereby overcoming target mutations and cofactor‑mediated resistance; and (3) the implementation of precision biomarker panels, including Merlin IHC, YAP/TAZ nuclear scoring, and circulating *CTGF*/*CYR61* monitoring, to enable patient stratification and real‑time pharmacodynamic assessment.

#### 6.3.1 Next‐Generation Inhibitor Design

##### 6.3.1.1 Subtype Selectivity

Exploiting the tissue‐specific expression and functional differences among TEAD1–4 isoforms are critical to minimizing normal‐tissue toxicity. TEAD1‐sparing inhibitors are essential for cardiovascular and chronic fibrotic indications. Although the PBPs of TEAD1–4 are highly similar, small structural differences such as those leveraged by MSC‐1254 and MSC‐5046 to achieve TEAD1 selectivity are exploitable [138]. For chronic fibrosis, TEAD4‐selective degraders have shown preclinical efficacy in pulmonary fibrosis models while sparing TEAD1‐expressing cardiac tissues [139].

##### 6.3.1.2 Direct YAP or TAZ Inhibitors

All clinical‐stage inhibitors to date target TEAD. Developing small molecules that directly bind and inhibit YAP or TAZ would permit functional discrimination between the two paralogs and could modulate TEAD‐independent YAP/TAZ functions. However, this remains a formidable chemical challenge, given the extensive intrinsically disordered regions in YAP/TAZ proteins.

##### 6.3.1.3 PROTAC and Molecular Glue Optimization

The physicochemical properties of PROTACs and molecular glues require optimization to enhance oral bioavailability, brain penetration (for meningioma and schwannoma), and membrane permeability. The success of amine 1 (MW ∼655 Da) [143] demonstrates that glue‐like degraders can achieve substantially lower molecular weights than traditional PROTACs while retaining potent degradation activity.

#### 6.3.2 Rational Combination Strategies

A comprehensive array of mechanism‑guided combinations has been proposed to address the resistance nodes identified above, as systematically cataloged in Figure 5. These range from vertical pathway blockade, such as combining TEAD inhibitors with KRAS/MEK inhibitors to quench AP‑1 feedback, or with EGFR‑TKIs to eradicate drug‑tolerant persisters, to horizontal strategies that engage the immune system (TEAD inhibition plus anti‑PD‑1/PD‑L1). The combination of TEAD inhibitors with MAPK pathway antagonists. The AP‐1/FOSL1 feedback activation induced by GNE‐7883 [197] and the synergistic activity of IAG933 with KRAS^G12C^ inhibitors [115] provide a strong rationale for this combination. Enhanced AP‐1 transcription factor activity and restored YAP/TEAD chromatin occupancy jointly mediate resistance to the allosteric pan‐TEAD inhibitor GNE‐7883, and MAPK inhibitors reduce FOS like 1 (FOSL1) expression, sensitizing resistant cells both in vitro and in vivo [197]. Additionally, combination of IAG933 with the KRAS^G12D^ inhibitor MRTX1133 produced benefit in KRAS^G12D^‐mutant PDAC lines and PDX [115]. Consistently, IAG933 plus osimertinib conferred enhanced antitumor benefit and led to rapid regression in the EGFR‐mutated NCI‐H1975 NSCLC xenograft model [115]. Early clinical evaluation in KRAS‐mutant NSCLC and CRC is warranted. The combination of TEAD inhibitors with CDK4/6 inhibitors or chemotherapy. In MPM, K‐975 demonstrated synergistic antitumor effects with cisplatin and pemetrexed chemotherapy, with the combination group achieving the greatest survival benefit compared with monotherapy or vehicle in orthotopic xenograft models [125]. K‐975 also exerted synergy with the CDK4/6 inhibitor palbociclib in subcutaneous mesothelioma mouse models. These combinations may counteract cell‐cycle‐mediated resistance. Sequential versus concurrent dosing. The optimal dosing sequence and ratios for these combinations have not been systematically evaluated. Prospective clinical trials incorporating pharmacodynamic and pharmacokinetic analyses are needed. Immune checkpoint inhibitors (ICI) combinations. Given that TEAD inhibition suppresses PD‐L1 and CD24 [177, 179], combination with anti‐PD‐1/PD‐L1 antibodies is mechanistically attractive, but requires careful management of T‐cell‐intrinsic YAP/TAZ effects, potentially through tumor‐selective delivery or intermittent scheduling.

To translate these mechanistically plausible combinations into viable regimens, several clinical‐realism considerations must be addressed explicitly. Overlapping toxicities are a central constraint. Combining TEAD inhibitors with MEK or EGFR inhibitors compounds gastrointestinal and dermatologic toxicity [200, 210], combination with CDK4/6 inhibitors adds hematologic toxicity [211], and pairing with chemotherapy or immune‐checkpoint inhibitors raises the risk of cumulative organ and immune‐related adverse events. Tolerability, rather than the efficacy alone, therefore often dictates feasible doses [130, 137, 212, 213]. The first‐in‐human trial of the TEAD palmitoylation inhibitor VT3989 (*n* = 172) showed a safety profile dominated by grade 1–2 events (proteinuria, peripheral edema, fatigue), with proteinuria proving reversible upon dose adjustment, further reinforcing the primacy of the tolerability boundary in dose selection [130]. In addition, scheduling matters. Concurrent dosing can maximally suppress adaptive resistance but is often intolerable; sequential or pulsed/intermittent regimens, for instance TEAD‐inhibitor priming followed by a targeted agent, or alternating cycles, may preserve efficacy while widening the therapeutic window [47, 137]. The optimal sequence should be defined pharmacodynamically rather than assumed. Furthermore, patient selection should be biomarker‐anchored, for instance restricting KRAS‐ or EGFR‐inhibitor combinations to tumors with demonstrable YAP/TAZ‐driven adaptive resistance, recent genome‐wide CRISPR screens have repeatedly identified Hippo pathway genes among the top hits in EGFR‐TKI persister cells and lung cancer patient‐derived organoid resistance models, providing causal‐level evidence for this enrichment strategy [157, 161]. Similarly, immunotherapy combinations should focus on immune‐excluded, YAP/TAZ‐high tumors [47, 210]. Finally, pharmacodynamic biomarkers (TEAD target‐gene signatures, circulating *CTGF*/*CYR61*, and on‐treatment YAP/TAZ nuclear scoring) should be embedded prospectively to confirm dual target engagement, guide dose and schedule, and detect emerging resistance [208, 214, 215]. In short, combinations should be developed as biomarker‐defined, pharmacodynamically‐scheduled regimens rather than as empirical drug pairings [47, 137].

#### 6.3.3 Biomarker‐Driven Precision Medicine

The disappointing ORR of 0% for IK‐930 in unselected NF2‐mutant patients underscores that NF2 mutation status alone constitutes an insufficient predictive biomarker. Future clinical development should therefore embrace multiparameter patient stratification encompassing Merlin protein expression assessed by immunohistochemistry, YAP/TAZ nuclear localization status, TEAD target gene signatures including CTGF and CYR61, and VGLL4/VGLL3 expression levels, with the Merlin–YAP dual immunohistochemistry assay developed for VT3989 representing an important step in this direction [155]. Pharmacodynamic biomarkers should incorporate dynamic changes in circulating CTGF and CYR61 or intratumoral gene signatures as early indicators of target engagement and pathway suppression. Single‐cell profiling is warranted to resolve YAP/TAZ activity across distinct tumor subpopulations, CAF subsets and immune infiltrates, thereby enabling more precise prediction of combination therapy benefit. Resistance monitoring through liquid biopsy approaches should be implemented to detect emergent TEAD mutations, VGLL3 upregulation or MAPK pathway activation during treatment, potentially enabling early intervention before clinical progression.

#### 6.3.4 Expanding the Therapeutic Window

Tissue‐specific delivery strategies represent promising but largely unexplored directions, with fibroblast activation protein‐activated prodrugs for fibrosis, ADCs for tumor‐selective delivery, and lung epithelial‐directed nanoformulations for IPF warranting further investigation. Intermittent and chronotherapeutic dosing schedules merit optimization based on clinical experience with VT3989, as such approaches can reduce renal and gastrointestinal toxicity while preserving antitumor efficacy. Whether YAP/TAZ activity in normal tissues follows circadian rhythms has not yet been explored. Conditional degraders including photo‐controlled or hypoxia‐inducible PROTACs could enable spatiotemporally precise TEAD degradation, thereby confining therapeutic effects to the tumor microenvironment or fibrotic lesion.

#### 6.3.5 Clinical Translation and Therapeutic Applications Beyond Oncology

Clinical translation of TEAD inhibitors for IPF, liver fibrosis and cardiac fibrosis requires careful patient selection, validated tissue‐specific endpoints and mitigation of regenerative impairment, with TEAD4‐selective inhibitors or FAP‐activated prodrugs potentially representing particularly suitable approaches for pulmonary fibrosis. In cardiovascular disease, validating whether the phenotypes of cardiac fibroblast‐specific YAP/TEAD deletion can be recapitulated by TEAD2/3/4‐selective inhibitors constitutes a critical step toward clinical development, given that the essential role of TEAD1 in CMs necessitates absolute TEAD1 sparing [174, 175]. In regenerative medicine, developing small molecules capable of transient, local YAP/TAZ activation through LATS inhibitor prodrugs or photo‐activatable compounds holds transformative potential for promoting tissue repair in aging and injury, though the dosing window must be rigorously controlled to mitigate oncogenic risk and targeted delivery or local administration strategies are essential [146]. For perioperative management, standardized protocols for temporary drug discontinuation are needed for patients on chronic TEAD inhibitor therapy who require surgery, guided by the pharmacokinetic half‐life of the specific agent and the anticipated kinetics of wound healing.

## 7 Conclusion

The YAP–TEAD signaling axis has emerged as a therapeutically tractable node in the Hippo pathway, with multiple small‐molecule inhibitors now advancing through early‐phase clinical trials rather than having reached mature clinical validation. The Phase 1/2 trial of VT3989 demonstrated objective responses in NF2‐altered mesothelioma, establishing proof‐of‐concept for TEAD‐targeted therapy in genetically selected populations. In parallel, next‐generation compounds such as vinyl sulfone pan‐TEAD inhibitors, PROTAC degraders and molecular glues continue to expand the chemical space and offer mechanistically distinct approaches that may overcome the limitations of current inhibitors. Resistance to TEAD inhibition is multifactorial, encompassing MAPK pathway activation, VGLL cofactor switching, metabolic adaptation and drug efflux among other mechanisms. These findings underscore that monotherapy is unlikely to produce durable responses in most patients. Rational combination strategies particularly with KRAS and EGFR pathway inhibitors will be essential. Equally important, the therapeutic window of pan‐TEAD inhibitors is constrained by on‐target toxicities in normal tissues, motivating the development of subtype‐selective agents and targeted delivery systems.

The next wave of progress will likely come from several directions. Mechanistically distinct modalities including molecular glue and proteolysis targeting chimera degraders exemplified by KG‐FP‐003 and amine 1 may overcome intrinsic resistance and achieve more durable pathway suppression. Meanwhile, upstream kinase modulators, nanobody‐based YAP degraders and RNA‐ and gene‐based therapeutics are beginning to broaden the druggable landscape of the pathway. Furthermore, companion diagnostics that integrate Hippo pathway mutation status, YAP/TAZ nuclear localization, Merlin expression and VGLL cofactor levels will be critical to guide patient selection and combination strategies. Additionally, transient, spatiotemporally precise modulation of YAP–TEAD signaling, whether via selective LATS inhibitors or conditional small molecules, holds transformative potential for regenerative medicine, provided that the risks of sustained activation can be rigorously controlled. Finally, the expansion of TEAD‐targeted therapy beyond oncology into fibrosis and cardiovascular disease will require a renewed focus on tissue‐specific targeting and paralog‐selective pharmacology.

Realizing the full therapeutic promise of the Hippo pathway will require the continued convergence of structural biology, medicinal chemistry, mechanism‐based translational research, and rigorous clinical investigation. The journey from an “undruggable” PPI to a clinically tractable target class has been remarkable; the path from here to truly precision‐guided, tissue‐specific Hippo pathway modulation represents the next frontier. As illustrated by IAG933, IK‐930, and VT3989, clinical success is determined by mechanism, paralog selectivity, and dosing schedule, not by target engagement alone. More critically, safety should be incorporated into the initial design. Given the physiological roles of YAP/TAZ–TEAD, TEAD1‐sparing selectivity, reversible engagement, and tissue‐restricted delivery are not incremental refinements but prerequisites for chronic use, particularly in nononcology indications. Notably, patient selection should evolve from single‐gene criteria such as *NF2* status toward composite, dynamically monitored biomarker panels. Such panels, incorporating Merlin and YAP/TAZ nuclear status, *VGLL* cofactor levels, and circulating *CTGF*/*CYR61*, should anticipate resistance and be prospectively embedded in trial design. Prospectively, TEAD‐directed therapy is well positioned to mature from proof‐of‐concept into a durable, precision‐guided, and mechanistically diversified therapeutic class spanning oncology, fibrosis, and regenerative medicine.

## Author Contributions

**Xiaodan Qu**: investigation, visualization, formal analysis, writing – original draft, supervision, writing – review and editing, funding acquisition. **Zhan‐you Wang**: supervision, writing – review and editing. All the authors have read and approved the final version of the manuscript.

## Funding

This work was supported by the grants from National Natural Science Foundation of China (Grant No. 82304635).

## Ethics Statement

The authors have nothing to report.

## Conflicts of Interest

The authors declare no conflicts of interest.

## Data Availability

The authors have nothing to report.
